# Defining Genome-Wide Expression and Phenotypic Contextual Cues in Macrophages Generated by Granulocyte/Macrophage Colony-Stimulating Factor, Macrophage Colony-Stimulating Factor, and Heat-Killed Mycobacteria

**DOI:** 10.3389/fimmu.2017.01253

**Published:** 2017-10-03

**Authors:** Samer Bazzi, Emale El-Darzi, Tina McDowell, Helmout Modjtahedi, Satvinder Mudan, Marcel Achkar, Charles Akle, Humam Kadara, Georges M. Bahr

**Affiliations:** ^1^Faculty of Science, Engineering and Computing, School of Life Sciences, Kingston University, Kingston upon Thames, United Kingdom; ^2^Faculty of Sciences, University of Balamand, Al Kurah, Lebanon; ^3^Faculty of Medicine and Medical Sciences, University of Balamand, Al Kurah, Lebanon; ^4^Department of Translational Molecular Pathology, The University of Texas MD Anderson Cancer Center, Houston, TX, United States; ^5^St George’s University of London, Imperial College, London and The Royal Marsden Hospital, London, United Kingdom; ^6^Clinical Laboratory, Nini Hospital, Tripoli, Lebanon; ^7^Immodulon Therapeutics Ltd., Uxbridge, United Kingdom; ^8^Faculty of Medicine, Department of Biochemistry and Molecular Genetics, American University of Beirut, Beirut, Lebanon

**Keywords:** monocyte-derived macrophages, heat-killed mycobacteria, *Mycobacterium obuense*, RNA sequencing, CD molecules, cytokines, chemokines

## Abstract

Heat-killed (HK) *Mycobacterium obuense* (NCTC13365) is currently being evaluated in the clinic as an immunotherapeutic agent for cancer treatment. Yet, the molecular underpinnings underlying immunomodulatory properties of HK *M. obuense* are still largely undefined. To fill this void, we sought to perform immunophenotyping, chemokine/cytokine release analysis and genome-wide characterization of monocyte-derived macrophages (MDM) in which monocytes were originally isolated from healthy donors and differentiated by HK *M. obuense* (Mob-MDM) relative to macrophage colony-stimulating factor (M-MDM) and granulocyte/macrophage colony-stimulating factor (GM-MDM). Immunophenotyping and cytokine release analysis revealed downregulated surface expression of CD36, decreased spontaneous release of CCL2 and increased spontaneous secretion of CCL5, CXCL8/IL-8, IL-6, and TNF-α in Mob-MDM relative to M-MDM and GM-MDM. Analysis of cytostatic activity showed that Mob-MDM exhibited similar growth inhibitory effects on immortalized and malignant epithelial cells compared with GM-MDM but at an elevated rate relative to M-MDM. To understand global cues in Mob-MDM, we performed comparative RNA-sequencing (RNA-Seq) analysis of Mob-MDM relative to GM-MDM and M-MDM (*n* = 4 donors). Clustering analysis underscored expression profiles (*n* = 256) that were significantly modulated in Mob-MDM versus both M-MDM and GM-MDM including, among others, chemokines/cytokines and their receptors, enzymes and transcriptions factors. Topological functional analysis of these profiles identified pathways and gene sets linked to Mob-MDM phenotype including nitric oxide production, acute phase response signaling and microbe recognition pathways as well as signaling cues mediated by the proinflammatory cytokine, interferon-gamma, and the intracellular pattern recognition receptor, nucleotide-binding oligomerization domain-containing protein 2. Taken together, our study highlights molecular immune phenotypes and global signaling cues in Mob-MDM that may underlie immunomodulatory properties of HK *M. obuense*. Such properties could be of valuable use in immunotherapy approaches such as adoptive cell therapy against cancer.

## Introduction

Macrophages (Mφ) are key members of the mononuclear phagocytic system with fundamental roles in the development, repair and homeostasis of tissues (1, 2). Tissue Mφ are sustained through either local proliferation of cells or the recruitment of blood monocytes which in turn differentiate into Mφ (3, 4). Based on their activation/polarization state, Mφ are broadly classified into M1 and M2 types which represent the polar states of a functional continuum (5).

M1-Mφ, are proinflammatory, promote T-helper 1-type immune responses and possess strong antimicrobial and antitumor capacities. On the contrary, M2-Mφ, which are anti-inflammatory, promote Th2-type immune responses and support cell proliferation and tissue repair as well as contribute to angiogenesis and tumor progression (6–9). Accumulating evidence points to important roles for the microenvironment in modulating the phenotypic and functional heterogeneity of Mφ (10). Of note, macrophage colony-stimulating factor (M-CSF) and granulocyte/macrophage colony-stimulating factor (GM-CSF) are key growth factors involved in the development of monocyte-derived macrophages (MDM) (11, 12). Earlier reports suggest that unpolarized/steady-state Mφ developed *in vitro* in the presence of GM-CSF (GM-MDM) or M-CSF (M-MDM) would exhibit different phenotypes suggestive of M1-like or M2-like phenotypes, respectively (13–15). Generation of polarized GM-MDM (M1-Mφ) or M-MDM (M2-Mφ) *in vitro* requires additional stimulation with inflammatory stimuli/type 1 cytokine or with type 2 cytokines, respectively (16–18). Evidently, unpolarized M-MDM have been reported in various studies to result in Mφ which constitutively express selected genes, secrete certain cytokines/chemokines and/or express surface receptors that are quite distinct from those detected in unpolarized GM-MDM (14, 19–21). Despite the fact that GM-CSF was originally defined as a hematopoietic growth factor, several reports have demonstrated that monocytes differentiated into Mφ in the presence of GM-CSF (unpolarized GM-MDM) spontaneously release inflammatory cytokines/chemokines, whereas unpolarized M-MDM either do not release or release some of these molecules at significantly lower levels (16, 22, 23). Furthermore, the role of GM-CSF has been well described in several inflammatory diseases, including rheumatoid arthritis and experimental autoimmune encephalomyelitis (24, 25).

Over the past two decades, there has been substantial interest in identifying novel immunomodulatory agents to treat cancer as well as chronic infectious, inflammatory, and autoimmune diseases. It is well established that mycobacteria exert significant immunomodulatory effects (26, 27). For instance, bacillus Calmette–Guerin, an attenuated live strain of *Mycobacterium bovis*, induces potent Th-1 immune responses and has proven to be highly efficacious in the adjuvant treatment of non-muscle-invasive bladder cancer (28, 29). Another mycobacterium, *M. vaccae*, in the form of a heat-killed (HK) preparation, is able to correct Th-2/Th-1 imbalance through multifaceted effects such as stimulating cytotoxic T-cells (30), enhancing the antitumor activity of γδ T-cells (31) and downregulating Th-2 immune responses (32–34). As such, the immunotherapeutic potential of HK *M. vaccae* has been evaluated in the pathological setting including allergy, tuberculosis (26, 35) and a range of cancers such as melanoma (36) and those of the prostate (37), lung (38, 39), and kidney (40). Recently, HK *M. obuense* (NCTC13365) preparation has garnered interest as a promising immunotherapeutic agent for cancer (41, 42). In a phase I clinical study, HK *M. obuense* was shown to be safe and well tolerated in patients with advanced stage melanoma (42). More recently, a randomized phase II study revealed that HK *M. obuense*, used as an adjunctive immunotherapeutic agent, was well-tolerated and resulted in significant improvement in the clinical outcome of patients with metastatic pancreatic cancer (41). Underlying these therapeutic properties may be the impact of HK *M. obuense* on various components of the innate immune system (31, 43); for example HK *M. obuense* augmented the cytotoxic activity of “innate-like” γδ T cells (31) as well as regulated the surface expression of various receptors on monocytes, neutrophils (43), and dendritic cells (DCs) (44).

While HK mycobacteria have offered promising clinical applications, the molecular cues they impinge on innate immune cells is not yet well defined. To fill this void, we sought, first, to assess whether HK *M. obuense* could induce monocyte differentiation into Mφ and second, to perform phenotypic and genome-wide surveys of monocytes differentiated into Mφ in the presence of HK *M. obuense* (Mob-MDM) as compared to M-MDM and GM-MDM. We characterized cell surface profiles and chemokine/cytokine release patterns in Mob-MDM some of which were shared with those of M-MDM and/or GM-MDM. Further, whole-transcriptome sequencing coupled with functional pathways and gene-gene network analysis delineated contextual expression profiles that are significantly linked to Mob-MDM, indicative of an overall augmented proinflammatory M1-like Mφ phenotype. Our findings point to global effects of HK *M. obuense* on the immunophenotype and transcriptome of human innate immune cells and, thus, offer new insights on the immunomodulatory properties of HK *M. obuense* that may be gauged for new immunotherapeutic strategies.

## Materials and Methods

### Antibodies and Reagents

Mouse antihuman fluorescein isothiocyanate (FITC)-conjugated CD40 (clone 5C3), CD64 (clone 10.1), CD195 (clone 2D7/CCR5), CD197 (clone 150503); phycoerythrin (PE)-conjugated CD36 (clone CB38), CD80 (clone L307.4), CD163 (clone GHI/61), CD206 (clone 19.2); PE-Cyanine 7 (Cy7)-conjugated CD16 (clone 3G8); peridinin chlorophyll protein complex (Per-CP)-conjugated CD14 (clone MφP9), HLA-DR (Clone L243); allophycocyanin (APC)-conjugated CD1a (clone HI149), CD32 (clone FLI8.26), CD86 (clone 2331), HLA-ABC (clone G46-2.6) antibodies as well as mouse FITC-conjugated IgG1 (clone MOPC-21), IgG2a (clone G155-178); PE-conjugated IgG1 (clone MOPC-21); Per-CP conjugated IgG2a (clone X39), IgM (clone G155-228); and APC-conjugated IgG1 (clone MOPC-21) isotype control antibodies were obtained from BD Biosciences. Sterile vials of HK *M. obuense* (NCTC13365) suspended in borate-buffered saline (pH 8.0) at 50 mg/ml were manufactured by BioElpida and applied at a final concentration of 30 µg/ml. M-CSF and GM-CSF were applied at final concentrations of 100 ng/ml (R&D Systems).

### Collection of Blood from Healthy Donors

Peripheral blood samples (200–250 ml) were obtained from healthy adult donors through the blood bank at Nini Hospital, Lebanon. All donors provided a written informed consent prior to participation in this study. Blood was collected in citrate-phosphate-dextrose-adenine containing blood collection bags and stored at room temperature for 1–2 h prior to usage. All of the procedures used in the present study were approved by the institutional review board at the University of Balamand and by the research ethics committee at the Faculty of Science, Engineering and Computing at Kingston University.

### Isolation of Monocytes and Generation of MDM

Peripheral blood mononuclear cells (PBMCs) were separated from blood using the standard Ficoll-Paque density gradient method as previously described (43). PBMCs were seeded into culture flasks at a density of 1.5 × 10^6^ cells/ml and incubated overnight at 37°C in a 5% CO_2_ humidified incubator. Later, cells were washed extensively and adherent monocytes were allowed to differentiate for 5 days into Mφ in complete RPMI growth medium [supplemented with 7.5% heat-inactivated pooled human AB serum (ZenBio), 2 mM l-glutamine, 100 U/ml penicillin, and 100 µg/ml streptomycin (Sigma)] in the presence of 100 ng/ml M-CSF, 100 ng/ml GM-CSF (16, 23) or 30 µg/ml HK *M. obuense* to generate M-MDM, GM-MDM, and Mob-MDM, respectively. The viability of MDM was ~80% as determined by the trypan blue dye exclusion method. MDM purity, as assessed by flow cytometry, was >85% with the remaining of cells consisting primarily of lymphocytes.

### Immunophenotyping of MDM

To analyze cell surface receptor expression, 1 × 10^5^ MDM were preincubated with 10% human serum AB for 20 min at 4°C to ensure effective Fc receptor blocking. MDM were then incubated with optimized concentrations of antigen-specific or matching isotype control antibodies for 25 min at 4°C in the dark, washed with cell wash solution (BD Biosciences) and finally resuspended in 1% paraformaldehyde solution (Sigma). A total of 10,000 MDM were analyzed by a FACSCalibur flow cytometer (BD Biosciences) using the Cell Quest Pro software (BD Biosciences). Viable MDM were gated based on their side scatter (SSC) and forward scatter (FSC) properties. Cell surface receptor expression was reported as the percentage and as the mean fluorescence intensity (MFI) of receptor-positive MDM.

### Quantification of Chemokines, Cytokines, Growth Factors, and Nitric Oxide

Adherent MDM were washed extensively with cold PBS and detached by incubation in cold RPMI and gentle scraping. MDM were seeded in 24-well culture plates at a density of 3 × 10^5^ cells/well/ml and cultured in complete RPMI growth medium for 24 h at 37°C in a 5% CO_2_ humidified incubator. For the quantification of total nitrite levels, RPMI medium was replaced by phenol red-free DMEM/F12 medium (Sigma). After 24 h, MDM culture supernatants were collected and stored at −80°C for later analysis. Levels of human CCL2, CCL5, CCL22, CXCL8/IL-8, CXCL9, IL-6, IL-10, IL-12 (p40), IL-12 (p70), IL-23 (p19/p40), M-CSF, TGF-β1, TNF-α, total nitric oxide (total nitrite), and VEGF were quantified in MDM cell culture supernatants using commercially available ELISA kits and total nitric oxide kit following the manufacturer’s procedures (R&D Systems).

### Assessment of MDM Cytostatic Activity

HaCaT (kindly provided by Dr. Julnar Usta, American University of Beirut, Lebanon) or BxPC3 (kindly provided by Dr. Androulla Elia, St. George’s University of London, UK) cells were seeded in a 96-well plate at a density of 2.5 × 10^3^ and 5 × 10^3^ cells/well, respectively, and were allowed to adhere for 1 h at 37°C in a 5% CO_2_ humidified incubator. MDM were then cocultured with HaCaT or BxPC3 target cells for 48 h at an effector:target (E:T) cell ratio of 4:1 or 20:1 at 37°C in complete RPMI growth medium in a 5% CO_2_ humidified incubator. Cocultures (carried out in quadruplicates) were pulsed with 0.5 μCi/well of methyl-tritiated [3 H]-thymidine (Perkin Elmer) during the final 18 h of coculture. Cells were then harvested onto glass fiber filters (Connectorate AG) and the radioactivity of incorporated 3 H-thymidine was determined by a liquid scintillation counter (Perkin Elmer) and expressed as counts per minute (cpm). The data were normalized by subtracting the cpm of MDM cultured alone from the cpm of HaCaT- or BxPC3-MDM-cocultures. Results were reported as percentage of HaCaT or BxPC3 growth inhibition calculated as follows: [1 − (cpm of HaCaT- or BxPC3-MDM-cocultures/cpm of HaCaT or BxPC3 cells cultured alone)] × 100.

### Total RNA Isolation

Total RNA was purified from MDM using the RNeasy Plus kit (Qiagen) according to the manufacturer’s instructions. Concentrations of RNA samples were quantified using the NanoDrop 2000 spectrophotometer (Thermofisher) according to the manufacturer’s protocol. Quality was assessed by computing RNA integrity numbers using the Agilent 2100 Bioanalyzer according to the manufacturer’s instructions.

### Preparation of Whole-Transcriptome Libraries, Templates, and RNA-Sequencing (RNA-Seq)

Total RNA (800 ng) was ribosomal RNA depleted using the Low Input RiboMinus Eukaryote System v2 (Thermofisher) according to the manufacturer’s instructions. Samples were then vacuum concentrated and whole-transcriptome and barcoded libraries were prepared using the Ion Total RNA-Seq Kit v2 (Thermofisher) according to the manufacturer’s instructions. Size distribution of samples was assessed using the Agilent 2100 Bioanalyzer and 6000 RNA Pico kit. Library concentrations were quantified using the Agilent DNA 1000 assay and the 2100 Bioanalyzer according to the manufacturer’s instructions. Barcoded whole-transcriptome libraries were diluted to a 66 pM concentration and combined in equal volumes (13 µl each, 26 µl total) for sequencing with two samples per template preparation. All template reactions were performed on the Ion Chef Instrument using the Ion PI Hi-Q Chef kit (Thermofisher) according to the manufacturer’s instructions. Reactions were loaded onto Ion PI Chips v3 (Thermofisher) for sequencing on an Ion Proton sequencer according to the manufacturer’s protocol.

### RNA-Seq Analysis

Alignment of reads was performed using Partek flow and a two-step alignment procedure. Unaligned reads were first aligned (hg19) using the STAR algorithm (45). Following the first alignment, any unaligned reads were then realigned with Bowtie2 version 2.1.0 (46). Reads from both alignment steps were then combined. Transcripts were then quantified using a modified version of the expectation-maximization (E/M) algorithm as described previously (47). Resultant reads per kilobase per million (RPKM) values were first processed by adding a pseudocount to all values with RPKM < 1.0 followed by log (base 2) transformation and quantile normalization.

A fixed-effects models with ANOVA was used to identify transcripts significantly differentially expressed (*n* = 965, Table S1 in Supplementary Material) among GM-MDM, M-MDM, and Mob-MDM (*P* < 0.05 and twofold change thresholds). Analysis was performed in the R language environment and using the BRB-Array tools developed by Richard Simon and the BRB-ArrayTools development team (48). Select transcripts were also assessed in pair-wise comparisons (e.g., GM-MDM compared to M-MDM) using *t*-tests with random variance models. Unsupervised hierarchical clustering analysis was performed to identify, among the differentially expressed genes, those with different patterns of expression among the three MDM (49, 50). Functional pathways analysis, including gene set enrichment and gene-gene network analysis, of differentially expressed transcripts was performed using Ingenuity Pathways Analysis as described previously (51). Raw data were deposited into the gene expression omnibus under dataset series GSE102492 (samples GSM2739484 to GSM2739495).

### Statistical Analysis

Statistical analysis of data was performed using GraphPad Prism software (version 6; GraphPad Software). Data were presented as mean values ± SEM values. Statistical significance of differences between different MDM types was determined by one-way ANOVA test followed by the Tukey’s *post hoc* test for confirmation of significant differences in means among the different groups. *P* values <0.05 denoted statistical significance.

## Results

### Phenotypic Characterization of M-MDM, GM-MDM, and Mob-MDM

**Figure 1:**
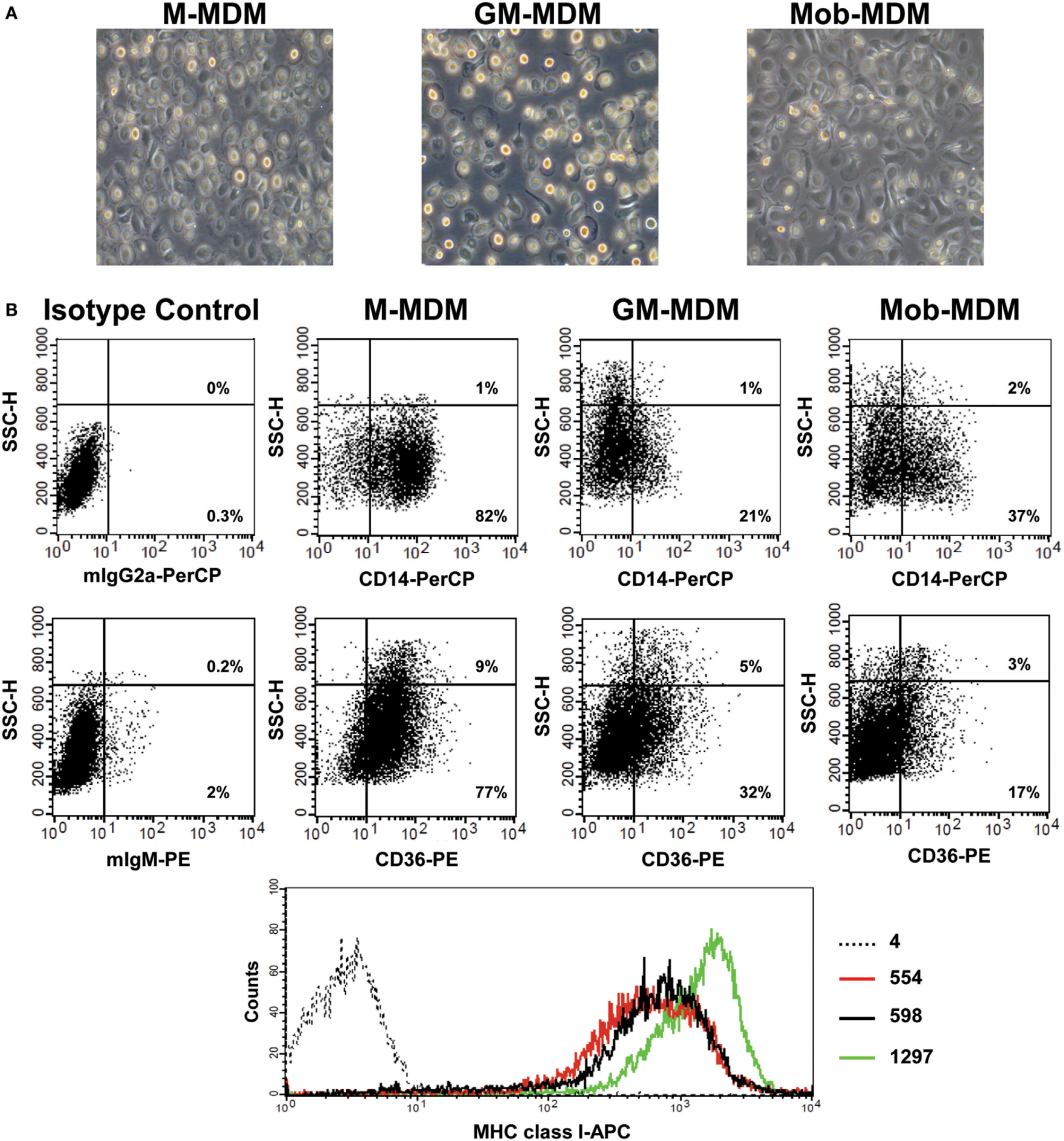
Morphological and immunophenotypic characterization of monocyte-derived macrophages (MDM). Macrophage colony-stimulating factor MDM (M-MDM), granulocyte/macrophage colony-stimulating factor MDM (GM-MDM), and *Mycobacterium obuense* MDM (Mob-MDM) were generated as described above (see Materials and Methods). **(A)** The morphology of the three MDM types was analyzed using a light-phase microscope (200× magnification). **(B)** Representative flow cytometry plots from one healthy donor showing the surface expression of CD14, CD36 (presented as dot plots), and MHC class I (presented as histogram plots) on different MDM types (black dotted line histogram: isotype control; red line histogram: GM-MDM; black line histogram: Mob-MDM; green line histogram: M-MDM). Numbers within lower and upper right quadrants of dot plots correspond to the percentage of receptor-positive MDM out of the total MDM population. Numbers next to histogram plots indicate the geometric mean fluorescence intensity (MFI) of receptor-positive MDM. SSC-H, side scatter height.

**Figure 2:**
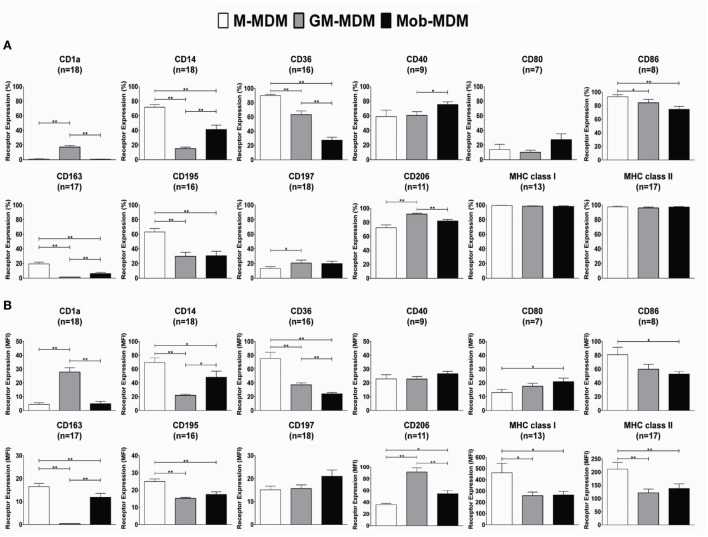
Expression levels of surface receptors on monocyte-derived macrophages (MDM). Macrophage colony-stimulating factor MDM (M-MDM), granulocyte/macrophage colony-stimulating factor MDM (GM-MDM), and *Mycobacterium obuense* MDM (Mob-MDM) were generated as described above (see Materials and Methods). The expression levels of select cell surface receptors were measured by flow cytometry. Column bars represent mean values of the **(A)** percentage (%) and **(B)** geometric mean fluorescence intensity (MFI) of receptor-positive MDM from at least seven independent healthy donors. Error bars represent SEM. Statistically significant differences in receptor expression among the MDM were determined by one-way ANOVA followed by the Tukey’s *post hoc* test (*P < 0.05; **P < 0.01).

We observed the morphology of different MDM generated by the disparate methods of monocyte-to-Mφ differentiation. M-MDM, GM-MDM, and Mob-MDM predominantly exhibited a round-shaped morphology with few spindle-shaped cells coexisting in cultures (Figure 1A). We then sought to compare and contrast the expression levels of various prototypic cell surface receptors on M-MDM, GM-MDM, and Mob-MDM. This group of surface receptors was selected on the basis of previous studies that described these validated receptors as selective markers for unpolarized/steady-state human M-MDM and GM-MDM (13, 16). Figure 1B shows representative flow cytometry plots analyzing the differential expression of CD14, CD36, and MHC class I on different MDM types. Our analysis revealed that M-MDM displayed higher surface expression levels (% and MFI of positive cells) of the M2-like markers CD14, CD36, CD163, and CD195, relative to GM-MDM and Mob-MDM, which both exhibited comparable levels of CD195 (Figures 2A,B). In contrast, Mob-MDM exhibited the lowest surface expression levels of CD36, whereas GM-MDM exhibited the lowest surface expression levels of CD14 and CD163 (Figures 2A,B). Of note, a small subset of GM-MDM (20%) expressed the DC marker, CD1a (52), which was expressed at extremely low levels (<1%) on both M-MDM and Mob-MDM (Figure 2A). Additionally, we noted that CD206 expression was significantly (*P* < 0.05) higher in GM-MDM compared to M-MDM and Mob-MDM (Figures 2A,B). We also probed the expression levels of other types of cell surface receptors. Although the percentage of MHC class I^+^ and MHC class II^+^ cells were similar among the three MDM types (Figure 2A), M-MDM exhibited significantly increased MFIs of both receptors (*P* < 0.05) cells compared to GM-MDM and Mob-MDM (Figure 2B). Also, M-MDM displayed significantly (*P* < 0.05) higher percentage of cells positive for the costimulatory marker, CD86 (Figure 2A). On the other hand, Mob-MDM exhibited the highest percentage of cells positive for the costimulatory/activation marker, CD40 (Figure 2A). It is worthwhile to note that we found no significant differences in the percentage of cells positive for the other costimulatory marker, CD80, or for the chemokine receptor 7, CD197, between the three MDM types (Figures 2A,B). We also studied the expression of different Fc receptors on M-MDM, GM-MDM, and Mob-MDM, generated from a smaller number of donors (*n* = 5). Statistically significant differences were reflected by a lower CD16 expression (% of positive cells) in Mob-MDM versus M-MDM, a higher CD64 expression (% of positive cells) in Mob-MDM versus GM-MDM, and a higher CD32 expression (% and MFI of positive cells) in M-MDM as compared to GM-MDM (Figure S1 in Supplementary Material). Our findings point to differential expression of prototypic cell surface markers in MDM differentiated in the presence of HK *M. obuense* relative to MDM generated by GM-CSF and M-CSF.

### Chemokine and Cytokine Profiles of M-MDM, GM-MDM, and Mob-MDM

**Figure 3:**
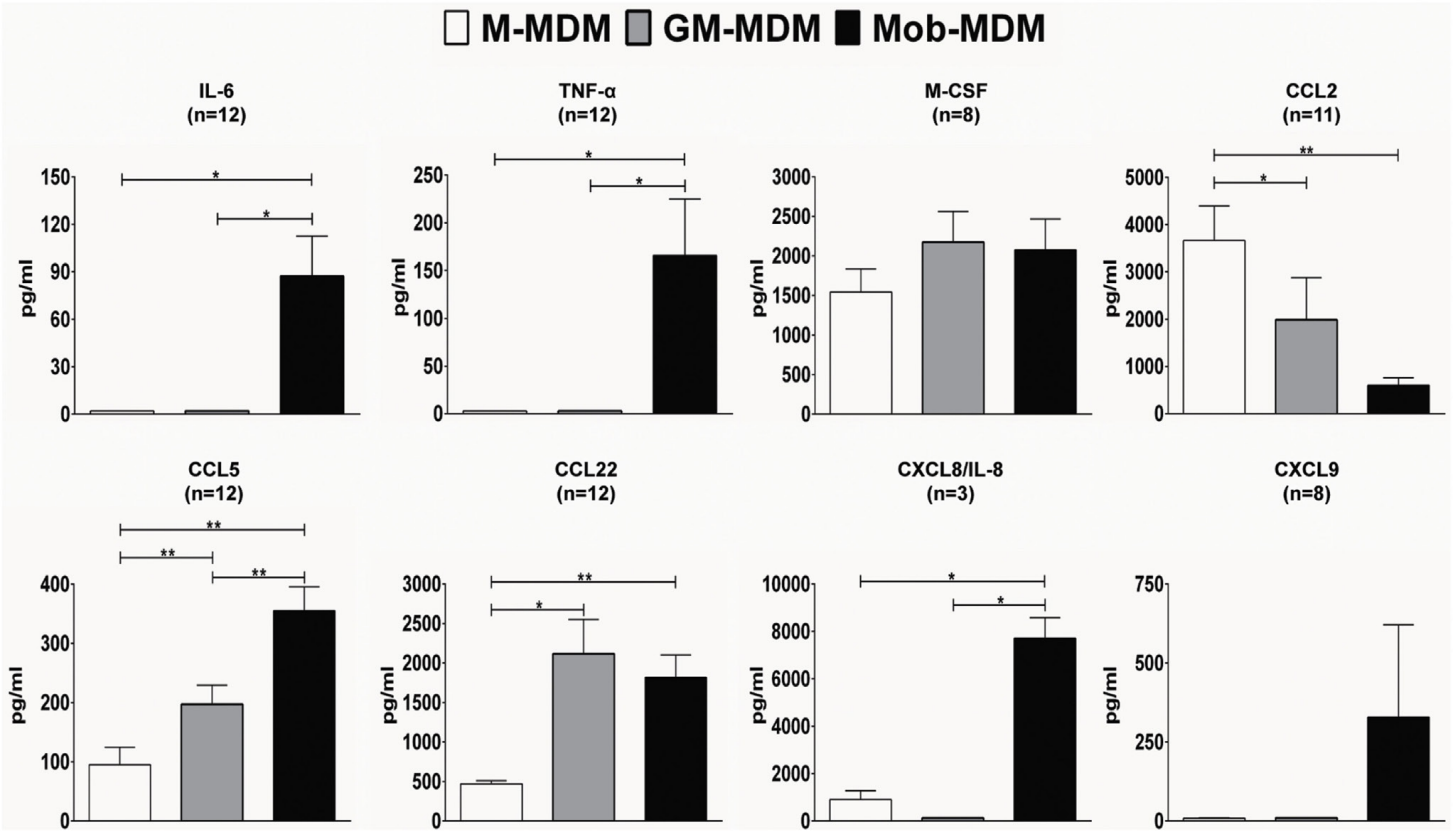
Chemokine and cytokine release by macrophage colony-stimulating factor monocyte-derived macrophages (M-MDM), granulocyte/macrophage colony-stimulating factor MDM (GM-MDM), and *Mycobacterium obuense* MDM (Mob-MDM). M-MDM, GM-MDM, and Mob-MDM were generated as described above (see Materials and Methods). Chemokine and cytokine levels were determined in MDM culture supernatants by ELISA. Column bars represent mean values of chemokine or cytokine concentration in MDM culture supernatants of at least three healthy independent donors. Error bars represent SEM. Statistically significant differences in chemokine/cytokine release levels were determined by one-way ANOVA followed by the Tukey’s *post hoc* test (*P < 0.05; **P < 0.01).

We next evaluated the profiles of a panel of spontaneously released proinflammatory and anti-inflammatory chemokines and cytokines, over a 24 h culture period, in M-MDM, GM-MDM, and Mob-MDM cultures. Both GM-MDM and Mob-MDM exhibited relatively higher levels of M-CSF compared with M-MDM, albeit not reaching statistical significance (Figure 3). We also noted that the levels of the chemokine CCL22 were significantly higher in both GM-MDM and Mob-MDM relative to M-MDM (4.5- and 3.8-fold, respectively; Figure 3). Unlike M-MDM and GM-MDM, Mob-MDM secreted significant (*P* < 0.05) levels of the proinflammatory cytokines, IL-6, TNF-α, and CXCL8/IL-8 (Figure 3). Of note, while CCL2 was lowest in Mob-MDM and highest in M-MDM, CCL5 exhibited reciprocal patterns of expression, i.e., the chemokine was significantly highest in Mob-MDM and lowest in the M-MDM type. Also, levels of secreted CXCL9 were only detected in supernatants of Mob-MDM, albeit CXCL9 levels were found to originate from only two out of the eight donors (Figure 3). Additionally, we did not detect IL-10, IL-12 (p40), IL-12 (p70), IL-23 (p19/p40), TGF-β1, and VEGF in M-MDM, GM-MDM, and Mob-MDM supernatants (data not shown). We then examined nitric oxide (NO) production through measuring its stable end-product nitrite in MDM culture supernatants. This analysis revealed that there were no detectable levels of nitrite found in culture supernatants of the three MDM types (data not shown). Our data strongly point to chemokine/cytokine profiles and immune phenotypes that are associated with Mob-MDM relative to both M-MDM and GM-MDM.

### Differential Cytostatic Activities of M-MDM, GM-MDM, and Mob-MDM

**Figure 4:**
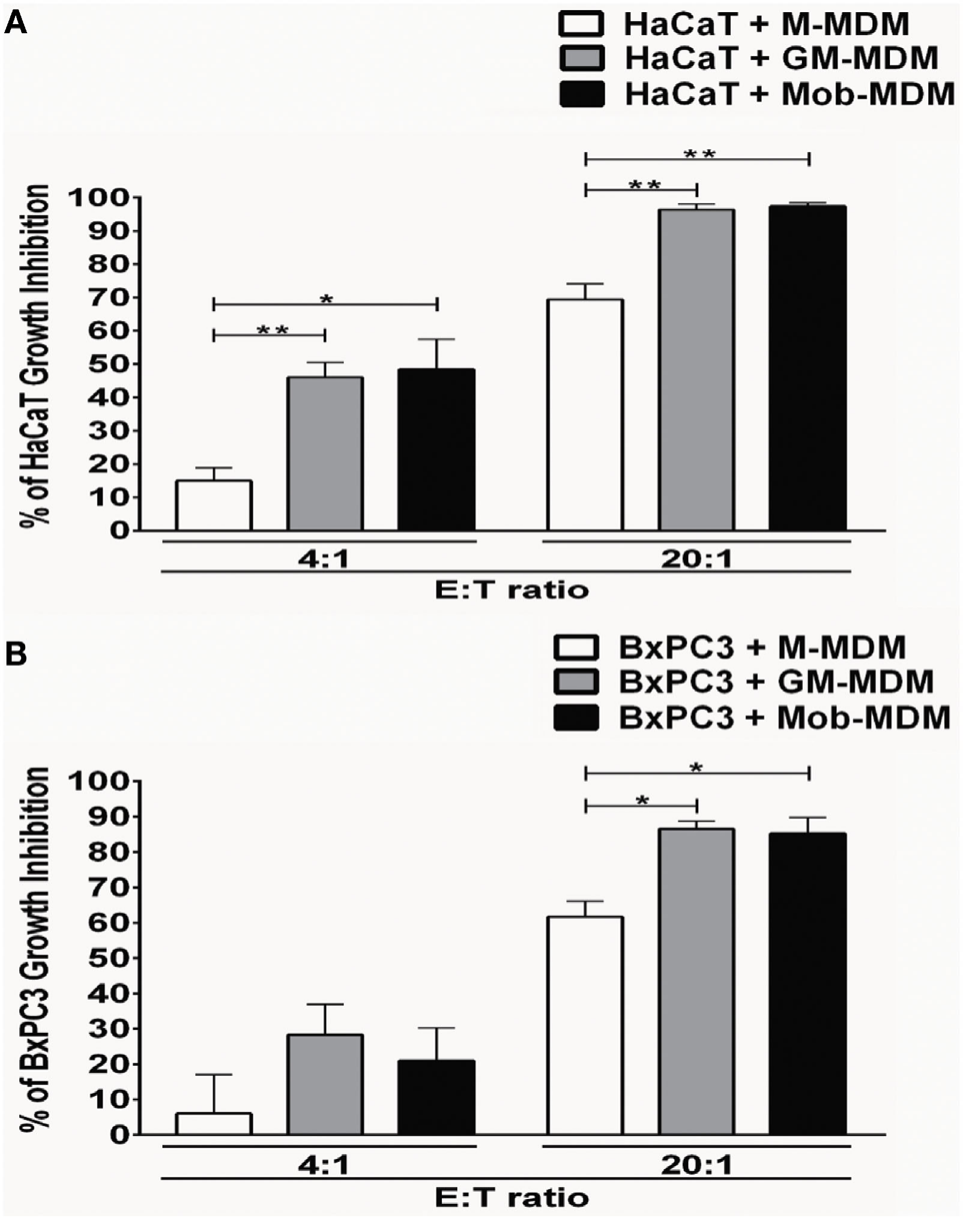
Analysis of antiproliferative effects of the monocyte-derived macrophages (MDM) on immortalized and malignant epithelial cells. Macrophage colony-stimulating factor MDM (M-MDM), granulocyte/macrophage colony-stimulating factor MDM (GM-MDM), and *Mycobacterium obuense* MDM (Mob-MDM) were generated as described above (and see Materials and Methods). The MDM were cocultured with HaCaT **(A)** or BxPC3 **(B)** cells at effector to target (E:T) cell ratios of 4:1 and 20:1 for 48 h. Cell proliferation was next determined by tritiated [3H]-thymidine incorporation assay and data were expressed as counts per minute (cpm). Percentage (%) of cell growth inhibition was calculated as described in the Section “Materials and Methods.” Column bars represent mean values of the % of cell growth inhibition induced by MDM from five **(A)** and four **(B)** independent healthy donors, respectively. Each experiment employing MDM from different donors were performed in quadruplicates. Error bars represent SEM. Statistically significant differences in cytostatic activity were determined by one-way ANOVA test followed by the Tukey’s *post hoc* test (*P < 0.05; **P < 0.01).

We next examined the cytostatic activities of M-MDM, GM-MDM, and Mob-MDM against the immortalized human keratinocyte cell line, HaCaT and the human ductal pancreatic adenocarcinoma cell line, BxPC3. Cell growth inhibition of both HaCaT and BxPC3 cells was apparent after 48 h of coculture with the different MDM types at 4:1 effector:target (E:T) ratio and became more pronounced at 20:1 (E:T) ratio (Figures 4A,B). At both E:T ratios, GM-MDM and Mob-MDM demonstrated similar growth inhibitory effects on HaCaT cells whereby these effects were significantly higher (*P* < 0.05) than those exerted by M-MDM (Figure 4A). At 4:1 (E:T) ratio, there were no significant differences in BxPC3 growth inhibition among M-MDM, GM-MDM, and Mob-MDM, whereas at 20:1 (E:T) ratio, there was significantly elevated growth inhibition of BxPC3 cells cocultured with GM-MDM and Mob-MDM relative to M-MDM (Figure 4B).

### Comparative RNA-Seq Analysis of Mob-MDM, M-MDM, and GM-MDM

**Figure 5:**
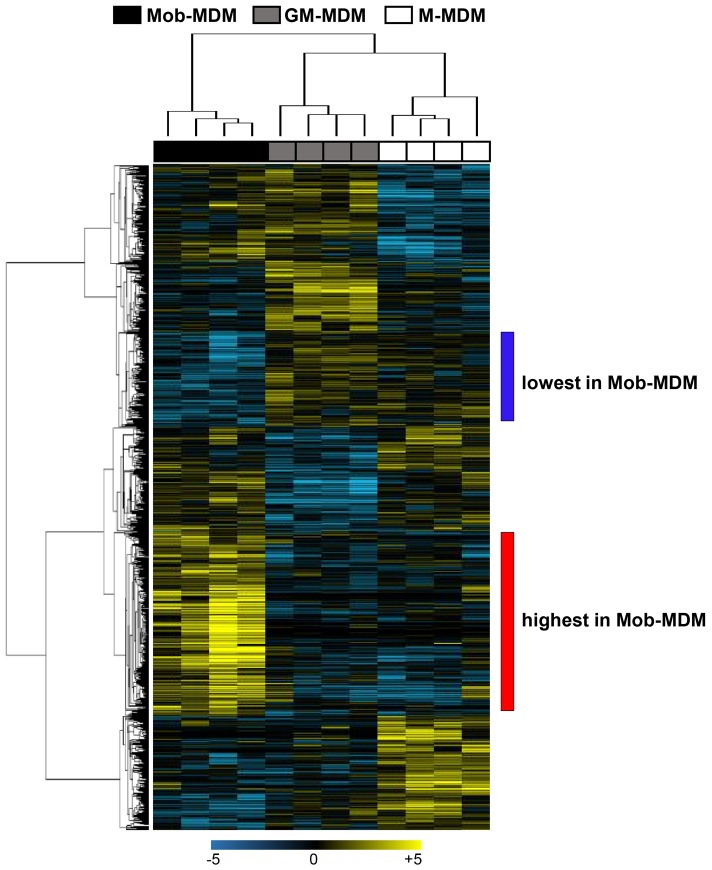
Whole-transcriptome analysis of monocyte-derived macrophages (MDM). Macrophage colony-stimulating factor MDM (M-MDM), granulocyte/macrophage colony-stimulating factor MDM (GM-MDM), and *Mycobacterium obuense* MDM (Mob-MDM) were generated as described above (and see Materials and Methods) from four healthy donors (*n* = 12). Total RNA was isolated and then sequenced using the Ion Torrent platform (as described in the Section “Materials and Methods”). Sequence alignment followed by quantification of transcriptomes was performed as described in the Section “Materials and Methods.” Transcripts (*n* = 965) differentially expressed between the three MDM were identified based on a fixed-effects model with ANOVA and analyzed by hierarchical clustering. Columns represent samples and rows constitute the differentially expressed transcripts (yellow, upregulated compared with the median sample; blue, relatively downregulated).

Our findings on the differential cell surface receptor and chemokine/cytokine profiles as well as tumor cell cytostatic activity of Mob-MDM relative to MDM-MDM and GM-MDM prompted us to compare and contrast genome-wide expression between the three MDM groups. We performed RNA-Seq, using the Ion Torrent Proton platform, of Mob-MDM, M-MDM, and GM-MDM derived from four donors (*n* = 12 samples). On average, we sequenced approximately 36 million reads per sample and achieved 90% uniformity of coverage. Following alignment and transcriptome quantification, we employed a fixed effects model and ANOVA with a statistical threshold of *P* < 0.05 and a twofold change cutoff to identify transcripts that were differentially expressed among the three groups (*n* = 965, Table S1 in Supplementary Material). Of the 965 transcripts, we confirmed markers that are known (19, 20) to be differentially expressed between M-MDM and GM-MDM including the transmembrane receptors *CD36, CD163, MRC-1* (*CD206*), and *STAB1*, the cytokine *TNFSF13*, the chemokine *CCL2*, the peptidase *ADAMDEC1* and others such as *SEPP1* surface receptors (Table S2 in Supplementary Material). We then performed unsupervised hierarchical clustering analysis to delineate subgroups or clusters of genes with differential patterns of expression amongst the three MDM. Two-dimensional hierarchical clustering of both transcripts and samples revealed that Mob-MDM clustered separately from both M-MDM and GM-MDM (Figure 5). Of note, this analysis revealed two clusters of transcripts that exhibited lowest and highest relative expression in Mob-MDM compared with both M-MDM and GM-MDM (Figure 5). Among the two clusters, further analysis revealed 256 transcripts that were differentially expressed in Mob-MDM relative to both M-MDM and GM-MDM (Table S3 in Supplementary Material).

**Figure 6:**
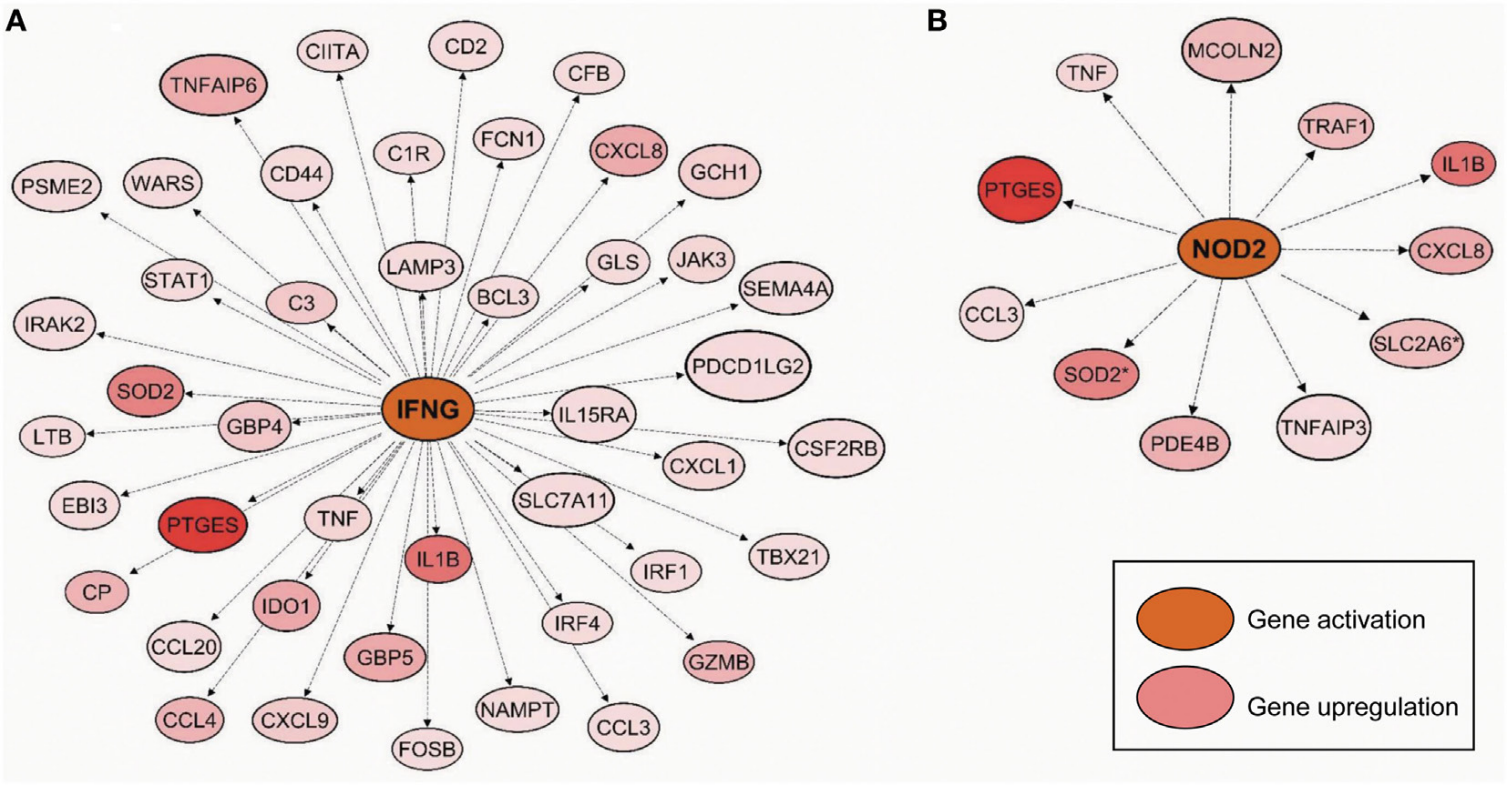
Predicted activated gene-gene networks mediated by interferon-gamma (*IFNG*) and nucleotide-binding oligomerization domain-containing protein 2 (*NOD2*) in *Mycobacterium obuense* monocyte-derived macrophages (Mob-MDM). Macrophage colony-stimulating factor MDM (M-MDM), granulocyte/macrophage colony-stimulating factor MDM (GM-MDM), and Mob-MDM were generated as described above (and see Materials and Methods) from four healthy donors (*n* = 12) and profiled by RNA-seq. Transcripts (*n* = 256) that were significantly differentially expressed in Mob-MDM relative to both M-MDM and GM-MDM, were interrogated by pathways, gene–gene network and gene set enrichment analyses using the commercially available software Ingenuity Pathways Analysis (IPA). Gene–gene network analysis demonstrated predicted activation (indicated by the orange color) of **(A)** interferon-gamma (*IFNG*) and **(B)** nucleotide-binding oligomerization domain-containing protein 2 (*NOD2*) along with upregulation (red color) of downstream genes in the *IFNG*- and *NOD2*-mediated networks in Mob-MDM relative to both GM-MDM and M-MDM.

Following the differential expression and clustering analysis, we were prompted to understand functional pathways and gene sets that are linked to Mob-MDM. Gene ontology analysis revealed that the Mob-MDM-associated transcripts comprised chemokines/cytokines, enzymes such as peptidases, phosphatases, and kinases as well as transmembrane receptors and transcription regulators (Table S3 in Supplementary Material). Select chemokines and cytokines, namely CXCL8/IL-8 and TNF-α, were confirmed by ELISA to be expressed in Mob-MDM (Figure S2 in Supplementary Material). Pathways analysis underscored canonical signaling pathways that were significantly modulated in Mob-MDM relative to M-MDM and GM-MDM including nitric oxide signaling, acute phase response, TREM1, and IL-6 (all *P* < 0.05; Table S4 in Supplementary Material). Gene set enrichment analysis predicted significant activation or inhibition (*z*-score ≥ 2 or ≤−2, respectively) of signaling modules in Mob-MDM relative to both GM-MDM and M-MDM but not between the latter two MDM types (all *P* < 0.05; Table S5 in Supplementary Material). These included marked activation of cytokines/chemokines such as interferon-gamma *(IFNG, z*-score = 4.98) and of intracellular receptors such as nucleotide-binding oligomerization domain-containing protein 2 (*NOD2, z*-score = 3.14) with *P* values < 0.0001 (Figure 6). The gene set and network analysis also underscored biological functions such as increased differentiation of mononuclear leukocytes (*z*-score = 3.0), elevated movement of mononuclear leukocytes (*z*-score = 4.41), and phagocytes (*z*-score = 4.24) that were significantly modulated (all *P* < 0.0001) in Mob-MDM relative to both GM-MDM and M-MDM but not among the latter two MDM (Table S6 in Supplementary Material). Our coupled RNA-Seq and functional pathways analysis point to functional gene expression programs in Mob-MDM that may underlie context-specific immunomodulatory effects of HK *M. obuense*.

## Discussion

Heat-killed preparations of the mycobacterium *M. obuense* have demonstrated promise in the immunotherapeutic setting in the clinic (41). However, there is much paucity in our knowledge either in the mechanisms of HK *M. obuense* as a driver of monocyte to Mφ differentiation or in its potential role in driving the polarization of human Mφ toward the M1-Mφ phenotype. Although it has been commonly reported that several bacteria can trigger the polarization of differentiated Mφ toward the M1-Mφ phenotype (53), other types of bacteria were reported to induce specific M2 programs (54). In addition, different mycobacterial species have been shown to exhibit differential effects on differentiated Mφ. While viable *M. tuberculosis* cultured with differentiated Mφ was found to induce the gene expression of IL-6 and TNF-α, viable *M. leprae* failed to induce significant changes in gene expression levels of either cytokine (55). Moreover, the effects of viable *M. tuberculosis* on Mφ gene expression profiles were reported to be quite distinct from those induced by HK *M. tuberculosis* (56). To our knowledge, the present study is the first to demonstrate the ability of HK *M. obuense* to induce the differentiation of human monocytes into Mφ. Moreover, it provides the first phenotypic and genome-wide whole transcriptome characterization of Mob-MDM in comparison to unpolarized/steady-state M-MDM and GM-MDM. Our results underscored surface receptor and cytokine protein profiles that accentuate the immunophenotype in Mob-MDM relative to the other two Mφ types. Transcriptome profiling by RNA-Seq, coupled with functional pathways analysis, pointed to activated (by virtue of gene expression) gene sets and networks indicative of an overall augmented proinflammatory M1-like Mφ phenotype in Mob-MDM relative to both M-MDM and GM-MDM. Our integrative study, probing for both protein and transcript profiles, provides molecular insights that may underlie immunomodulatory effects of HK *M. obuense* and that may be used as markers of response to HK *M. obuense*-based immunotherapy.

In the present study, M-MDM and GM-MDM revealed distinct expression patterns of a group of surface receptors which were concordant with those reported in previous studies (13, 16, 17, 57, 58). We noted significant differences in receptor expression profiles between M-MDM and GM-MDM with the former Mφ comprising elevated levels of CD14, CD32, CD163, and MHC class II and the latter Mφ depicting augmented CD206 expression. On the other hand, our data, as well as others (13, 18, 59), demonstrated that M-MDM and GM-MDM exhibit similar expression patterns of another group of surface receptors comprising CD16, CD40, CD64, and CD86. Nevertheless, these findings were not in agreement with other reports (16, 58, 60) and such discrepancies in cell surface receptor expression patterns between M-MDM and GM-MDM might be attributed to variations in culture conditions adopted in different studies, and in particular the concentration of M-CSF and GM-CSF used as well as the duration of the differentiation period. It is important to note that we detected a subpopulation (~20%) in GM-MDM that expressed CD1a. This cell surface receptor is typically expressed on monocyte-derived DCs differentiated in the presence of GM-CSF and IL-4 (61). Of note, previous studies (16, 21) similarly demonstrated elevated CD1a expression on GM-MDM. In Mob-MDM, we noted markedly diminished surface expression of CD36, a class B scavenger receptor implicated in the uptake of oxidized low density lipoprotein, sensing of bacteria and clearance of apoptotic cells (62), when compared to M-MDM and GM-MDM. It is worthwhile to mention that CD36 expression was also lower in GM-MDM compared to M-MDM with the receptor being the highest in the latter Mφ. Our findings are consistent with previous studies suggesting CD36 to function as a prototypic M2-Mφ marker (63). Of note, previous studies have suggested proinflammatory cues mediated by CD36-mediated signaling (64, 65). Our immunophenotypic findings point to cell surface profiles in Mob-MDM that are indicative of proinflammatory signaling in these immune cells following differentiation by HK *M. obuense*.

Along with the immunophenotypic analysis, we assessed spontaneous cytokine release profiles of the three MDM types. In line with previous studies, M-MDM spontaneously secreted higher levels of CCL2 (13, 14, 19) and lower levels of CCL22 (13, 16) as compared to unpolarized GM-MDM. We did not detect any spontaneous production of IL-6, IL-12 (p40), IL-12 (p70), IL-23 (p19/p40), or TNF-α in unpolarized M-MDM and GM-MDM culture supernatants and this in agreement with previously published results (13–15, 18, 23, 58). Although IL-10 has been previously reported to be spontaneously released at extremely low levels by M-MDM (17, 23, 58), we and others (14, 15) did not detect any spontaneous IL-10 secretion by M-MDM. In addition, CXCL9, mainly secreted by polarized GM-MDM (M1-Mφ) (66), was not detected in unpolarized GM-MDM culture supernatants. Compared with M-MDM and GM-MDM, Mob-MDM exhibited significantly elevated spontaneous release of the proinflammatory cytokines IL-6 and TNF-α and chemokine CXCL8/IL-8. The present findings are in line with our previous report interrogating cytokine release in whole blood exposed to HK *M. obuense* (43). Together, these findings highlight the potential of HK *M. obuense* preparation to activate cells of the innate immune system, and suggest probable signaling molecules in mycobacteria that are casually linked to release of proinflammatory chemokines/cytokines in these cells. Indeed, mycobacterial-associated molecules, such as heat shock proteins and muramyl peptides, have been previously demonstrated to trigger multiple proinflammatory chemokines and cytokines (67–70). It is worthwhile to note that CXCL8/IL-8 was shown to be a potent neutrophil chemoattractant (71). In this context, it is plausible to surmise that Mob-MDM exhibit significant neutrophil chemoattractant properties, e.g., in an infection setting. An additional salient feature of Mob-MDM that we observed was release of attenuated CCL2 levels in comparison with M-MDM and GM-MDM. CCL2 is a chemokine shown to help promote Th2 polarization by dampening Mφ activation and proinflammatory cytokine production (72). In studies surveying neoplasm pathology, CCL2 levels are positively associated with tumor-associated Mφ that typically exhibit an M2-like phenotype (14, 73). Our findings render the supposition that, in sharp contrast to M-CSF and GM-CSF, HK *M. obuense* resulted in differentiated Mφ (Mob-MDM) with elevated cytokine release signatures indicative of a proinflammatory M1-like Mφ phenotype.

In this study, we found that both Mob-MDM and GM-MDM exhibited higher antiproliferative effects against human pancreatic cancer cells, BxPC3, when compared to M-MDM. Similar differential cytostatic activities of GM-MDM and M-MDM have been previously reported when evaluated against colorectal cancer cells (74). In contrast, both M-MDM and GM-MDM did not demonstrate antitumor cell cytostatic effects in previous reports studying different osteosarcoma cell lines (75). It is noteworthy, that we found similar antiproliferative effects by Mob-MDM and GM-MDM in immortalized keratinocyte epithelial cells (HaCaT) suggesting that the antitumor effects of such Mφ (Mob-MDM) may extend to the premalignant setting. Of note, the proinflammatory cytokine TNFα, which we found to be secreted preferentially by Mob-MDM, was previously reported to inhibit the proliferation of various pancreatic cancer cell lines *in vitro* (76, 77). It is reasonable to speculate that soluble factor(s) released by the Mφ (e.g., lymphotoxin-β and TNFα) may underlie the elevated cytostatic properties displayed by Mob-MDM and GM-MDM. However, it cannot be neglected that additional mechanisms may mediate the antiproliferative effects of Mob-MDM and GM-MDM, such as the interaction (and signaling thereof) between tumor cells and Mφ warranting future studies to probe this hypothesis. It is worthwhile to mention that a recent phase II clinical trial investigated the potential immunotherapeutic use, in combination with chemotherapy, of HK *M. obuense* in pancreatic cancer patients (41). In this reported clinical study by Dalgleish et al., HK *M. obuense* was shown to exhibit immunotherapeutic effects when given in combination with chemotherapy, in pancreatic cancer patients with metastatic disease, a patient subpopulation for which there are no current targeted/immunotherapeutic treatment strategies. Our mechanistic findings and the aforementioned recent clinical report underscore the promising potential of immunotherapeutic strategies utilizing HK *M. obuense*, for advanced malignancies.

We performed RNA-Seq analysis to compare and contrast genome-wide expression among the three MDM. Our sequencing analysis pointed to 965 transcripts that were significantly differentially expressed among Mob-MDM, GM-MDM, and M-MDM. Among those, profiles significantly modulated between M-MDM and GM-MDM comprised transcripts previously reported to be higher (e.g., *CD163* and *STAB1*) and lower (e.g., *CCL24* and *MRC1/CD206*) in M-MDM (19, 20) relative to GM-MDM. We also found 256 transcripts linked to Mob-MDM phenotype as they were not modulated between M-MDM and GM-MDM. These transcripts likely indicate that *M. obuense*-driven differentiation resulted in an Mφ phenotype that expresses significantly higher levels of certain markers (Table S3 in Supplementary Material), as compared to GM-MDM, that are frequently associated with classically activated M1-Mφ. Such markers include, among others, *TNF, IL-6, CCR7*, nicotinamide phosphoribosyltransferase (*NAMPT*), formyl peptide receptor 1 (*FPR1*), and chemokine-like receptor 1 (*CMKLR1*) (13, 14, 78–81). Moreover, the Mob-MDM selective transcripts included a battery of molecules which potentiate the antimicrobial activity of innate immune Mφ including, among others, several complement components, cytokines and chemokines, G protein-coupled receptors such as *FPR1* (82) and *CYP27B1*. *CYP27B1* encodes 25-hydroxyvitamin D3 1-α-hydroxylase which generates the bioactive form of 25-hydroxyvitamin D3, namely 1,25-dihydroxyvitamin D3. This metabolite activates vitamin D receptor, thereby inducing release of antimicrobial peptides (83) and triggering the antimicrobial activity of Mφ against *M. tuberculosis* (84).

Our RNA-Seq profiling coupled with pathways and gene-gene network analysis pointed to various topological functional gene networks predicted to be significantly and selectively modulated in Mob-MDM including, among others, gene-gene networks mediated by the proinflammatory cytokine, *IFNG* (85), and the intracellular pattern recognition receptor, nucleotide oligomerization domain 2 (*NOD2*). Notably, both the *IFNG*- and *NOD2*-mediated networks comprised proinflammatory markers including *IL1B, CXCL8*, and *TNF*. Previous work has shown that the NOD2 receptor recognizes the N-glycolyl muramyl dipeptide present in mycobacteria (86), thus implicating NOD2 as one of the candidate receptors that may be involved in mediating the immunomodulatory effects of HK *M. obuense*. It is worthwhile to mention, as indicated above, that we had found elevated expression of the protein products of *CXCL8* and *TNF* in Mob-MDM. Our orthogonal analysis provide supportive framework for the finding of proinflammatory gene profiles in Mob-MDM. Our gene set enrichment analysis also demonstrated that various toll-like receptors (TLRs) were significantly and selectively predicted to be activated in Mob-MDM. Previous studies have shed light on critical roles for innate cell TLRs in mediating recognition of and response to mycobacterial antigens (87, 88). For instance, TLR-2 (as a heterodimer with either TLR-1 or TLR-6) -4, and -9 have been shown to be engaged in sensing various mycobacterial cell wall components such as glycolipids, glycoproteins, lipoproteins, and unmethylated CpG motifs in mycobacterial DNA (87, 88). In addition, studies from our group have underscored crucial roles for TLR-1 and -2 in mediating HK *M. obuense*-induced modulation of surface receptor expression on human monocytes (43) and DCs (44). Differentiation of monocytes into macrophages has been previously associated with ligation and activation of certain TLRs including TLR-2/1 (89). This could explain, at least in part, the ability of HK *M. obuense* to drive monocyte differentiation into Mφ. Our coupled RNA-Seq and functional pathways analysis provide insights into transcriptional programs in Mob-MDM that resemble, but not restricted to, the classically–activated M1-like Mφ phenotype.

All in all, our integrative immunophenotypic and genome-wide transcriptomic study reveals cell surface profiles, chemokine/cytokine release patterns, gene expression profiles and gene-gene networks in Mob-MDM that are quite different from those observed in upolarized/steady-state GM-MDM and M-MDM. Our study also sheds light on contextual genome-wide signaling cues in Mob-MDM that accentuate how HK *M. obuense* may program innate immune cells toward an elevated proinflammatory M1-like Mφ phenotype. Thus, one potential implication of our findings would be the use of Mob-MDM in an immunotherapeutic approach such as adoptive cell transfer of macrophages to treat cancer patients. In fact, adoptive cellular immunotherapy in cancer patients using autologous macrophages generated *in vitro* from blood monocytes has been extensively reported, but with limited success (90–93). Our present study warrants future investigations to further probe the functional responses of Mob-, M-, and GM-MDM following activation with an inflammatory stimulus in the presence or absence of a type 1 cytokine.

## Ethics Statement

All donors provided a written informed consent prior to participation in this study. All of the procedures used in the present study were approved by the institutional review board at the University of Balamand and from the research ethics committee at the Faculty of Science, Engineering and Computing at Kingston University.

## Author Contributions

SB, HK, and GB conceived and designed the experiments. SB, EE-D, and TM performed the experiments. SB, HM, SM, CA, HK, and GB analyzed the data. HM, SM, MA, HK, and CA contributed reagents, materials, and analysis tools. SB, HK, and GB wrote the article. All authors approved the final manuscript.

## Conflict of Interest Statement

SM and CA are unsalaried directors and shareholders of Immodulon Therapeutics, Ltd. GB is a member of the Scientific Advisory Board for Immodulon Therapeutics, Ltd. The remaining authors declare no potential conflicts of interest with respect to the research, authorship, and/or publication of this article.
