# Supplementary material for: Defining Genome-Wide Expression and Phenotypic Contextual Cues in Macrophages Generated by Granulocyte/Macrophage Colony-Stimulating Factor, Macrophage Colony-Stimulating Factor, and Heat-Killed Mycobacteria
Source: Front Immunol. 2017 Oct 3;8:1253. doi: 10.3389/fimmu.2017.01253 (PMC5632758; doi:10.3389/fimmu.2017.01253)
Supplement: Supplementary file 1 [file Data_Sheet_1.pdf]

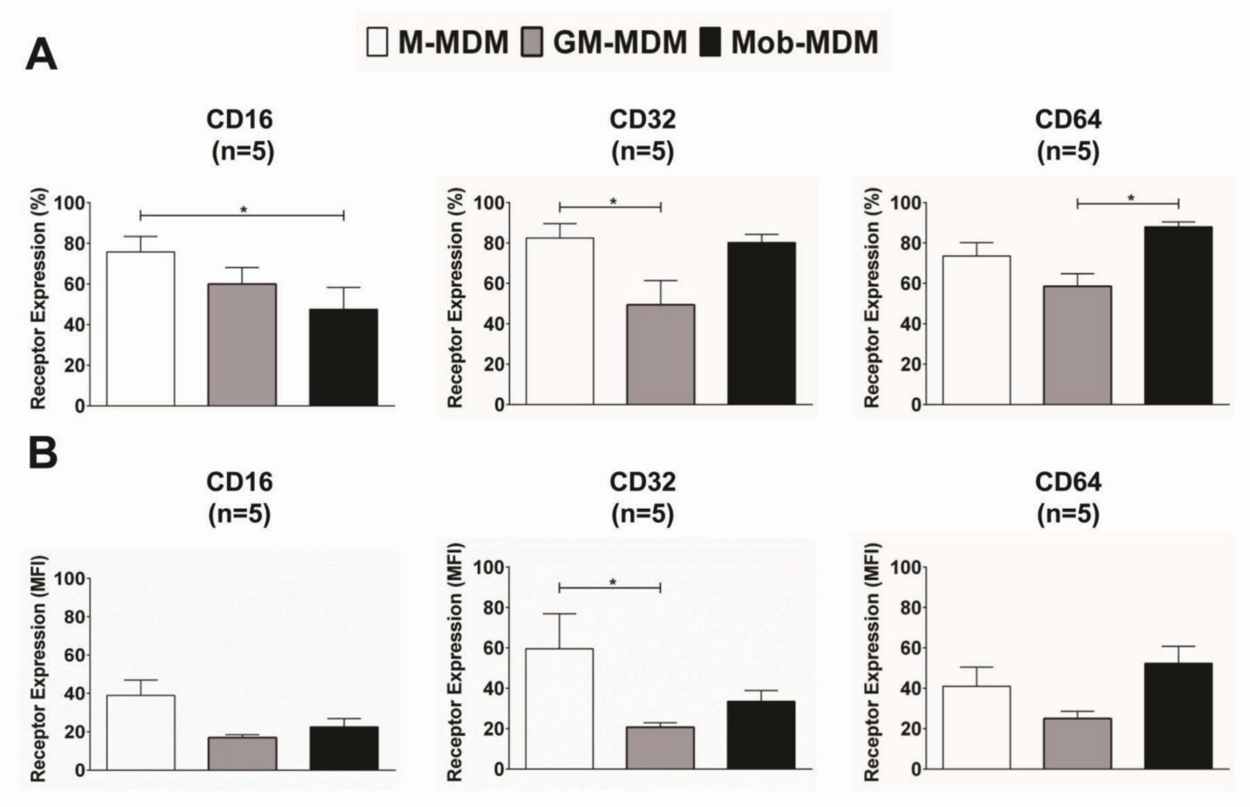

Supplementary Figure 1. Expression levels of surface receptors on monocyte-derived macrophages (MDM). Macrophage colony-stimulating factor MDM (M-MDM), granulocyte/macrophage colony-stimulating factor MDM (GM-MDM), and *Mycobacterium obuense* MDM (Mob-MDM) were generated as described in Materials and Methods. The expression levels of CD16, CD32 and CD64 were measured by flow cytometry. Column bars represent mean values of the (A) percentage (%) and (B) geometric mean fluorescence intensity (MFI) of receptor-positive MDM from 5 independent healthy donors. Error bars represent standard error of the mean (SEM). Statistically significant differences in receptor expression among the MDM were determined by one-way ANOVA followed by the Tukey's post-hoc test (*$P < 0.05$).

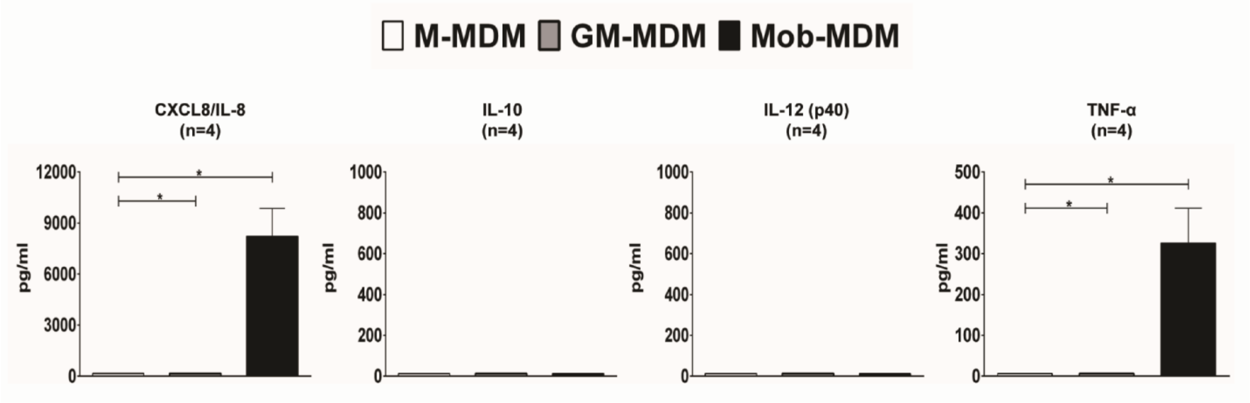

Supplementary Figure 2. Confirmation of select cytokines and chemokines identified to be differentially expressed among the monocyte derived macrophages (MDM) by RNA-sequencing. Macrophage colony-stimulating factor MDM (M-MDM), granulocyte/macrophage colony-stimulating factor MDM (GM-MDM), and *Mycobacterium obuense* MDM (Mob-MDM) were generated as described in Materials and Methods. The differential expression of select cytokine/chemokine transcripts (CXCL8/IL-8, IL-10, IL-12 and TNF-α) in the MDM was confirmed at the protein level by analysis of secreted levels in MDM culture supernatants by ELISA. Column bars represent mean values of cytokine or chemokine concentration in culture supernatants of the same MDM that were used in RNA-seq experiments and from the four independent healthy donors (see Materials and Methods section). Error bars represent standard error of the mean (SEM). Statistically significant differences were determined by one-way ANOVA followed by the Tukey's post-hoc test (*$P < 0.05$).

Supplementary Table 1. Transcripts significantly differentially expressed among M-MDM, GM-MDM and Mob-MDM

		Fold Change		
**RefSeq transcript ID**	**Symbol**	**Mob-MDM vs. GM-MDM**	**Mob-MDM vs. M-MDM**	**M-MDM vs. GM-MDM**
NM_014479	*ADAMDEC1*	37.36	4.14	9.03
NM_001823	*CKB*	34.23	11.52	2.97
NM_004878	*PTGES*	31.71	36.95	-1.17
NM_201442	*C1S*	31.38	40.36	-1.29
NM_001142343	*CMKLR1*	29.78	2.65	11.22
NM_015714	*G0S2*	25.73	18.89	1.36
NM_000576	*IL1B*	20.54	13.04	1.58
NM_001024465	*SOD2*	17.94	12.83	1.40
NM_014358	*CLEC4E*	15.32	5.97	2.57
NM_145640	*APOL3*	12.40	7.94	1.56
NM_002164	*IDO1*	12.19	12.19	1.00
NM_001134486	*GBP5*	12.01	10.60	1.13
NM_000584	*IL8*	11.89	22.67	-1.91
NM_007115	*TNFAIP6*	11.78	20.37	-1.73
NM_001135146	*SLC39A8*	11.70	1.05	11.20
NM_001037339	*PDE4B*	11.06	7.69	1.44
NM_002985	*CCL5*	10.80	4.67	2.31
NM_000096	*CP*	10.38	12.60	-1.21
NM_002984	*CCL4*	10.37	7.04	1.47
NM_004131	*GZMB*	10.34	10.34	1.00
NM_178833	*SLC9B2*	10.33	5.76	1.79
NM_178232	*HAPLN3*	9.89	9.89	1.00
NM_000878	*IL2RB*	9.85	5.38	1.83
NM_002982	*CCL2*	9.47	1.13	8.36
NM_001190945	*TRAF1*	9.47	2.87	3.30
NM_001190947	*TRAF1*	9.45	5.19	1.82
NM_000636	*SOD2*	9.43	6.19	1.52
NM_153259	*MCOLN2*	9.28	5.81	1.60
NM_002160	*TNC*	9.11	-2.23	20.32
NM_020530	*OSM*	9.08	4.02	2.26
NM_017585	*SLC2A6*	9.02	8.15	1.11
NM_002988	*CCL18*	9.00	17.35	-1.93
NM_001252392	*TNIP1*	8.96	5.32	1.68

NM_001135147	*SLC39A8*	8.84	1.44	6.13
NM_004530	*MMP2*	8.24	2.35	3.50
NM_005951	*MT1H*	8.21	6.33	1.30
NM_001001435.1	*CCL4L1*	8.17	5.48	1.49
NM_207007.1	*CCL4L2*	8.17	5.48	1.49
NM_033274	*ADAM19*	8.14	8.34	-1.02
NM_001193301	*SEMA4A*	7.88	1.59	4.96
NM_001024466	*SOD2*	7.81	4.84	1.61
NM_022367	*SEMA4A*	7.73	1.59	4.88
NM_001252391	*TNIP1*	7.73	4.89	1.58
NM_052941	*GBP4*	7.63	7.63	-1.00
NR_046450	*LOC374443*	7.56	5.45	1.39
NM_015549	*PLEKHG3*	7.54	6.83	1.10
NM_001193300	*SEMA4A*	7.54	1.51	4.98
NM_001838	*CCR7*	7.42	5.00	1.48
NM_138768	*MYEOV*	7.41	2.31	3.21
NM_001099287	*NIPAL4*	7.33	5.72	1.28
NR_003187	*NCF1C*	7.33	9.09	-1.24
NM_000064	*C3*	7.29	5.09	1.43
NM_002416	*CXCL9*	7.21	6.82	1.06
NM_001561	*TNFRSF9*	6.97	1.43	4.88
NM_004995	*MMP14*	6.87	4.50	1.53
NM_001955	*EDN1*	6.66	16.78	-2.52
NR_027835	*APOL3*	6.43	5.59	1.15
NM_000161	*GCH1*	6.43	7.98	-1.24
NM_031476	*CRISPLD2*	6.24	5.08	1.23
NM_005950	*MT1G*	6.19	6.03	1.03
NM_001001435	*CCL4L1*	6.07	4.61	1.32
NM_207007	*CCL4L2*	6.07	4.61	1.32
NM_022154	*SLC39A8*	6.05	1.39	4.35
NM_001165	*BIRC3*	6.01	2.18	2.76
NM_006378	*SEMA4D*	5.74	6.53	-1.14
NM_001017402	*LAMB3*	5.71	1.94	2.94
NM_000594.5	*TNF*	5.67	3.65	1.55
NR_003186	*NCF1B*	5.59	10.53	-1.88
NR_027833	*APOL3*	5.53	4.93	1.12
NM_005118	*TNFSF15*	5.48	1.43	3.84
NM_024812	*BAALC*	5.45	1.63	3.33
NM_001017995	*SH3PXD2B*	5.39	7.41	-1.37
NM_001145099	*SLC2A6*	5.37	6.30	-1.17
NM_021224	*ZNF462*	5.34	1.16	4.59

NM_144590	*ANKRD22*	5.33	7.93	-1.49
NM_000215	*JAK3*	5.26	6.37	-1.21
NM_000960	*PTGIR*	5.16	2.43	2.12
NM_001145271	*ADAMDEC1*	5.15	2.75	1.87
NM_001511	*CXCL1*	5.15	3.72	1.38
NM_000265	*NCF1*	5.13	8.45	-1.65
NM_021006	*CCL3L1*	5.04	5.37	-1.07
NM_000266	*NDP*	5.01	3.93	1.27
NM_005238	*ETS1*	4.99	2.74	1.82
NM_001130677	*C17orf96*	4.95	4.46	1.11
NR_022014	*HMGN2P46*	4.93	6.51	-1.32
NM_014331	*SLC7A11*	4.70	4.52	1.04
NM_002356	*MARCKS*	4.68	-1.49	6.97
NM_006041	*HS3ST3B1*	4.68	5.24	-1.12
NM_000022	*ADA*	4.65	1.13	4.10
NM_001733	*C1R*	4.64	8.03	-1.73
NM_004994	*MMP9*	4.63	1.24	3.73
NM_002122	*HLA-DQA1*	4.60	10.32	-2.24
NR_029417	*SHMT2*	4.56	1.71	2.67
NM_001142345	*CMKLR1*	4.55	4.39	1.04
NM_001111018	*NAV2*	4.54	3.01	1.51
NM_023009	*MARCKSL1*	4.53	1.92	2.36
NM_001767	*CD2*	4.50	4.01	1.12
NM_001169110	*SCO2*	4.48	6.23	-1.39
NM_001024858	*SPTB*	4.44	5.10	-1.15
NM_001193302	*SEMA4A*	4.35	3.09	1.41
NM_000675	*ADORA2A*	4.23	4.15	1.02
NM_015568	*PPP1R16B*	4.22	6.21	-1.47
NM_000607	*ORM1*	4.18	5.55	-1.33
NM_004273	*CHST3*	4.17	4.51	-1.08
NM_013309	*SLC30A4*	4.16	6.19	-1.49
NM_025092	*ATHL1*	4.11	1.89	2.18
NM_015441	*OLFML2B*	4.11	-2.75	11.27
NM_001271606	*BASP1*	4.03	3.10	1.30
NM_033105	*DNAJC5B*	3.98	3.39	1.17
NM_004603	*STX1A*	3.94	1.24	3.17
NM_021615	*CHST6*	3.91	3.91	1.00
NM_001258041	*HARS*	3.90	2.53	1.54
NM_001244950	*SPOCK2*	3.88	2.91	1.33
NM_001144966	*NEDD4L*	3.86	2.30	1.68
NM_006290	*TNFAIP3*	3.85	2.11	1.82

NM_001135775	*CACFD1*	3.85	4.81	-1.25
NM_020370	*GPR84*	3.84	1.57	2.44
NM_001570	*IRAK2*	3.78	2.20	1.72
NM_015393	*PARM1*	3.78	4.10	-1.08
NM_001734	*C1S*	3.78	10.78	-2.85
NM_030915	*LBH*	3.77	2.43	1.55
NM_024430	*PSTPIP2*	3.77	4.69	-1.24
NM_001080421	*UNC13A*	3.77	3.87	-1.03
NM_002185	*IL7R*	3.76	2.40	1.57
NM_017594	*DIRAS2*	3.75	3.91	-1.04
NM_005746	*NAMPT*	3.75	2.02	1.86
NM_182964	*NAV2*	3.74	1.58	2.37
NR_038319	*LOC100505716*	3.69	2.96	1.24
NM_001018071	*FRMPD2*	3.68	2.40	1.53
NM_002189	*IL15RA*	3.67	4.76	-1.29
NM_001033714	*NOP2*	3.65	-1.10	4.00
NM_130848	*C5orf20*	3.60	1.15	3.12
NM_001199241	*KYNU*	3.57	1.91	1.86
NM_013246	*CLCF1*	3.57	1.36	2.62
NM_006515	*SETMAR*	3.57	1.83	1.94
NM_001244960	*FRMD3*	3.53	3.38	1.04
NM_001136197	*FZR1*	3.51	1.64	2.14
NR_015384	*LOC100126784*	3.50	1.36	2.58
NM_020529	*NFKBIA*	3.47	1.72	2.02
NM_005098	*MSC*	3.45	3.06	1.13
NM_002198	*IRF1*	3.45	3.72	-1.08
NM_023072	*ZSWIM4*	3.42	1.74	1.97
NM_001025195	*CES1*	3.42	11.12	-3.25
NR_026880	*MGC12916*	3.42	3.85	-1.13
NM_005178	*BCL3*	3.39	2.29	1.48
NM_001098540	*HPSE*	3.38	1.01	3.36
NM_001124	*ADM*	3.36	1.11	3.04
NM_024508	*ZBED2*	3.33	3.06	1.09
NM_000127	*EXT1*	3.31	2.85	1.16
NM_001252385	*TNIP1*	3.29	3.57	-1.09
NM_001109	*ADAM8*	3.29	-1.18	3.89
NM_005755	*EBI3*	3.27	3.27	1.00
NM_001025194	*CES1*	3.25	15.78	-4.86
NM_000733	*CD3E*	3.23	2.52	1.28
NM_001270508	*TNFAIP3*	3.22	1.46	2.20
NM_001258456	*TNIP1*	3.22	2.25	1.43

NM_001270507	*TNFAIP3*	3.21	1.87	1.71
NM_001710	*CFB*	3.20	5.93	-1.85
NM_182508	*FAM216B*	3.18	4.43	-1.39
NM_002029	*FPR1*	3.18	2.54	1.25
NM_001142596	*P4HA1*	3.17	1.31	2.42
NM_005953	*MT2A*	3.15	4.67	-1.48
NM_152346	*SLC43A2*	3.14	1.31	2.40
NM_152386	*SGPP2*	3.12	3.12	1.00
NR_037840	*IL15*	3.11	1.98	1.57
NM_139266	*STAT1*	3.11	3.46	-1.11
NM_001270729	*BCL2L13*	3.10	3.01	1.03
NM_053056	*CCND1*	3.09	1.11	2.79
NM_015111	*N4BP3*	3.08	2.64	1.17
NM_001266	*CES1*	3.08	14.63	-4.75
NM_182757	*RNF144B*	3.05	2.38	1.28
NM_001039396	*MPEG1*	3.04	-1.23	3.73
NM_001244963	*NAV2*	3.03	-1.10	3.33
NM_002003	*FCN1*	3.01	3.16	-1.05
NM_001009186	*CCT6A*	3.00	1.45	2.07
NM_001243746	*FAM20A*	3.00	5.13	-1.71
NM_001007033	*CLEC6A*	3.00	5.62	-1.88
NM_001258455	*TNIP1*	2.98	2.26	1.32
NM_006795	*EHD1*	2.97	1.96	1.51
NM_001849	*COL6A2*	2.96	1.19	2.48
NR_037670	*SNX10*	2.95	2.90	1.02
NM_007315	*STAT1*	2.93	3.19	-1.09
NM_001199163	*PSMC5*	2.91	1.11	2.62
NM_022838	*ARMCX5*	2.91	2.53	1.15
NR_049793	*LOC100130476*	2.90	1.49	1.95
NM_006226	*PLCL1*	2.90	1.85	1.57
NM_152899	*IL4I1*	2.90	-1.10	3.18
NM_152866	*MS4A1*	2.83	3.78	-1.34
NM_198572	*SPATC1*	2.81	2.89	-1.03
NM_178507	*OAF*	2.80	1.16	2.41
NM_001127361	*RNF19B*	2.79	1.53	1.82
NM_001256765	*IL15RA*	2.79	2.62	1.06
NM_015973	*GAL*	2.78	-1.79	4.97
NM_001144073	*CHORDC1*	2.77	1.74	1.59
NM_004054	*C3AR1*	2.74	-1.02	2.80
NM_004267	*CHST2*	2.72	2.29	1.19
NM_017649	*CNNM2*	2.71	1.67	1.62

NM_032413	*C15orf48*	2.70	2.77	-1.03
NM_001013838	*RLTPR*	2.69	2.03	1.32
NM_001161819	*MYO1B*	2.68	3.17	-1.18
NM_003998	*NFKB1*	2.68	1.53	1.76
NM_001001438	*LSS*	2.68	2.23	1.20
NM_001195286	*IRF4*	2.68	2.62	1.02
NM_015149	*RGL1*	2.68	-1.77	4.76
NM_003507	*FZD7*	2.67	3.12	-1.17
NM_001077494	*NFKB2*	2.67	1.56	1.71
NM_000785	*CYP27B1*	2.67	2.30	1.16
NM_181357	*DCAF11*	2.66	-1.03	2.74
NR_023361	*AP3S2*	2.66	1.58	1.68
NM_001040657	*TTC23*	2.65	1.60	1.65
NM_152363	*ANKLE1*	2.64	2.26	1.17
NM_001008410	*STEAP3*	2.63	1.96	1.34
NM_001257231	*ALG13*	2.62	3.24	-1.24
NM_024873	*TNIP3*	2.61	2.61	1.00
NM_001164549	*DISC1*	2.60	1.29	2.01
NM_012307	*EPB41L3*	2.58	-1.14	2.93
NM_006139	*CD28*	2.58	2.40	1.07
NM_004195	*TNFRSF18*	2.56	2.44	1.05
NM_030813	*CLPB*	2.56	1.95	1.31
NM_014207	*CD5*	2.56	2.00	1.28
NM_001261410	*BCAR3*	2.55	1.65	1.54
NM_006779	*CDC42EP2*	2.52	2.50	1.01
NM_173701	*WARS*	2.52	3.73	-1.48
NM_001198786	*POU2F1*	2.51	2.57	-1.02
NM_152512	*ENTHD1*	2.50	2.55	-1.02
NM_014398	*LAMP3*	2.50	2.22	1.13
NM_006278	*ST3GAL4*	2.50	1.85	1.35
NM_000246	*CIITA*	2.49	4.04	-1.62
NM_002983	*CCL3*	2.47	2.87	-1.16
NM_016524	*SYT17*	2.47	1.36	1.81
NR_028076	*SCARF1*	2.46	2.17	1.13
NM_133174	*APBB3*	2.46	2.52	-1.02
NR_033781	*HSD3BP4*	2.45	1.94	1.26
NM_003546	*HIST1H4L*	2.44	1.09	2.24
NM_152320	*ZNF641*	2.43	3.94	-1.62
NM_175617	*MT1E*	2.43	3.39	-1.40
NM_001781	*CD69*	2.42	2.37	1.02
NM_001198812	*MSANTD3*	2.42	2.86	-1.18

NM_001628	*AKR1B1*	2.41	3.02	-1.26
NM_001144961.4	*NFKBIL1*	2.41	3.01	-1.25
NM_018234	*STEAP3*	2.40	1.77	1.36
NR_033319	*MIAT*	2.39	1.66	1.44
NM_207396	*RNF207*	2.38	5.21	-2.19
NM_001002810	*PDE4DIP*	2.38	1.86	1.28
NR_033149	*TMEM135*	2.37	1.24	1.92
NM_031281	*FCRL5*	2.37	2.01	1.18
NR_027767	*TNIK*	2.37	2.17	1.09
NM_000420	*KEL*	2.36	-1.73	4.09
NM_004479	*FUT7*	2.36	1.24	1.91
NM_001135047	*JDP2*	2.35	-1.70	4.01
NM_000130	*F5*	2.35	3.36	-1.43
NM_001199155	*KAT7*	2.34	1.18	1.98
NR_003945	*GVINP1*	2.34	2.78	-1.19
NM_001127704	*SERPINA1*	2.33	5.13	-2.20
NM_001164489	*ADAM8*	2.33	-1.34	3.12
NM_003581	*NCK2*	2.32	1.33	1.75
NR_045116	*C5orf56*	2.32	2.20	1.06
NM_001992	*F2R*	2.32	2.32	1.00
NM_003596	*TPST1*	2.32	-1.71	3.96
NM_006840	*LILRB5*	2.30	-4.76	10.97
NM_003811	*TNFSF9*	2.30	1.81	1.27
NR_024124	*ATP1A1OS*	2.30	1.05	2.20
NM_001260492	*RDX*	2.30	2.67	-1.16
NM_001025389	*AMPD3*	2.30	1.69	1.36
NM_080722	*ADAMTS14*	2.29	2.29	1.00
NM_000201	*ICAM1*	2.29	1.95	1.17
NM_003937	*KYNU*	2.28	1.81	1.26
NM_001146695	*KDM4C*	2.28	1.37	1.67
NR_045006	*NRON*	2.28	1.61	1.42
NM_002341.4	*LTB*	2.28	2.53	-1.11
NM_001161560	*TNIK*	2.27	2.54	-1.12
NM_025239	*PDCD1LG2*	2.27	2.32	-1.02
NM_002224	*ITPR3*	2.27	2.12	1.07
NM_005064	*CCL23*	2.26	2.69	-1.19
NM_021181	*SLAMF7*	2.26	1.60	1.42
NM_000358	*TGFBI*	2.26	-2.58	5.83
NR_034033	*LOC285972*	2.25	2.51	-1.11
NR_046035	*CXCL1*	2.25	2.07	1.09
NM_006806	*BTG3*	2.25	1.01	2.22

NM_001164440	*ANKRD33B*	2.25	2.85	-1.27
NR_045790	*NSUN4*	2.25	-1.39	3.11
NR_024420	*LOC389634*	2.24	2.86	-1.28
NM_001145000	*ITGAV*	2.23	1.24	1.81
NM_005937	*MLLT6*	2.23	1.37	1.63
NM_001018072	*BTBD11*	2.23	2.23	1.00
NM_006509	*RELB*	2.23	1.57	1.42
NM_001081638	*LILRB1*	2.22	1.37	1.62
NM_006080	*SEMA3A*	2.22	2.17	1.02
NM_006000	*TUBA4A*	2.21	1.30	1.70
NM_130470	*MADD*	2.20	1.62	1.36
NM_001256237	*PGAP2*	2.20	1.34	1.65
NR_037947	*NSUN2*	2.20	2.05	1.07
NM_013351	*TBX21*	2.19	2.56	-1.17
NM_001242348	*LOC100287177*	2.19	2.22	-1.01
NR_036436	*RSRC2*	2.18	1.98	1.10
NM_153047	*FYN*	2.17	-1.24	2.69
NM_001199417	*ARHGAP23*	2.17	1.14	1.91
NM_001195388	*FCRL5*	2.17	1.73	1.26
NM_016441	*CRIM1*	2.17	3.74	-1.73
NM_021136	*RTN1*	2.17	1.23	1.77
NM_002353	*TACSTD2*	2.17	3.57	-1.64
NM_000395	*CSF2RB*	2.17	2.69	-1.24
NM_006732	*FOSB*	2.16	2.13	1.02
NM_001142287	*SEMA4D*	2.16	1.97	1.10
NM_002818	*PSME2*	2.15	2.00	1.07
NM_001257198	*CUL3*	2.15	-1.83	3.93
NM_001001389	*CD44*	2.15	2.60	-1.21
NM_001099952	*ITPR1*	2.15	1.42	1.51
NR_034086	*LOC648987*	2.14	1.68	1.27
NM_033130	*SIGLEC10*	2.13	4.54	-2.13
NM_002340	*LSS*	2.13	1.75	1.22
NM_001261403	*NFKB2*	2.12	1.19	1.78
NM_001024024	*GCH1*	2.11	2.11	1.00
NM_022149	*MAGEF1*	2.11	1.21	1.74
NM_014323	*PATZ1*	2.11	2.40	-1.14
NM_001168272	*ITPR1*	2.11	1.84	1.14
NM_197955	*C15orf48*	2.10	2.25	-1.07
NR_033255	*DTYMK*	2.10	-1.04	2.18
NM_213602	*SIGLEC15*	2.09	1.10	1.91
NM_001130046	*CCL20*	2.08	2.08	1.00

NM_014905	*GLS*	2.08	2.18	-1.05
NM_001025390	*AMPD3*	2.07	1.23	1.68
NM_022143	*LRRC4*	2.06	1.34	1.54
NM_006564	*CXCR6*	2.06	1.68	1.23
NM_001002236	*SERPINA1*	2.06	7.39	-3.59
NM_001199837	*SNX10*	2.06	2.60	-1.26
NM_001135040	*DCTN1*	2.06	1.31	1.57
NM_001005353	*AK4*	2.05	2.44	-1.19
NM_001260506	*RPS3*	2.04	-1.07	2.19
NM_001136199	*GRAMD1A*	2.04	2.27	-1.11
NM_001145437	*LSS*	2.04	2.10	-1.03
NM_020128	*MDM1*	2.03	2.17	-1.07
NM_001258393	*CLPB*	2.03	1.57	1.29
NM_000593.6	*TAP1*	2.03	1.98	1.02
NR_036579	*APTX*	2.03	2.45	-1.21
NM_173653	*SLC9A9*	2.03	-1.03	2.10
NR_033652	*LOC100132891*	2.01	2.21	-1.10
NR_047645	*UTY*	2.01	1.77	1.14
NM_152496	*MANEAL*	2.01	2.88	-1.44
NM_000867	*HTR2B*	2.00	1.09	1.83
NM_001150	*ANPEP*	1.99	-1.34	2.66
NM_001142928	*LRRC61*	1.99	4.00	-2.01
NM_181079	*IL21R*	1.98	-1.48	2.95
NM_001017919	*RCCD1*	1.98	-1.43	2.83
NM_001184896	*PHF8*	1.98	-1.36	2.70
NM_013410	*AK4*	1.98	2.15	-1.08
NM_001145252	*CFP*	1.98	3.36	-1.70
NM_002197	*ACO1*	1.97	2.84	-1.44
NM_017565	*FAM20A*	1.97	2.19	-1.11
NM_001127218	*POLD2*	1.97	-1.33	2.62
NM_001265580	*FAM13A*	1.96	-1.67	3.28
NM_005086	*SSPN*	1.96	4.28	-2.18
NM_024637	*GAL3ST4*	1.96	-4.30	8.43
NM_001100818	*PID1*	1.95	-4.86	9.50
NM_172245	*CSF2RA*	1.95	2.30	-1.18
NM_005077	*TLE1*	1.95	2.28	-1.17
NM_005737	*ARL4C*	1.94	-4.32	8.37
NM_001946	*DUSP6*	1.93	-1.49	2.87
NM_000582	*SPP1*	1.93	-1.63	3.14
NM_005874	*LILRB2*	1.92	-1.12	2.16
NM_001170796	*ZFAND1*	1.89	2.22	-1.17

NM_001242916	*ZFAND6*	1.87	2.33	-1.24
NM_003178	*SYN2*	1.87	-2.15	4.03
NM_001040058	*SPP1*	1.87	-1.37	2.55
NM_138434	*C7orf29*	1.86	5.27	-2.83
NM_001195630	*MLLT10*	1.85	-1.18	2.18
NM_001256443	*PRRT2*	1.84	2.36	-1.28
NM_017522	*LRP8*	1.84	-1.09	2.01
NM_001141970.2	*DAXX*	1.84	2.30	-1.25
NM_001131055	*HRH2*	1.83	-1.40	2.56
NM_018176	*LGI2*	1.82	-3.40	6.19
NM_001198	*PRDM1*	1.82	-1.18	2.14
NM_144584	*HENMT1*	1.81	-1.23	2.24
NM_001114752	*CD55*	1.81	2.59	-1.43
NM_001012706	*C14orf182*	1.80	2.08	-1.16
NM_001001390	*CD44*	1.79	2.19	-1.22
NM_213645	*WARS*	1.78	3.25	-1.82
NM_002183	*IL3RA*	1.78	11.04	-6.20
NM_001260503	*SRP68*	1.77	-1.27	2.25
NM_005014	*OMD*	1.77	2.11	-1.20
NM_001267045	*PSME3*	1.76	-1.18	2.09
NM_002736	*PRKAR2B*	1.76	2.68	-1.52
NR_038414	*BZRAP1-AS1*	1.76	-1.47	2.59
NM_175058	*PLEKHA7*	1.76	2.42	-1.38
NR_026947	*C1RL-AS1*	1.75	4.66	-2.67
NM_016354	*SLCO4A1*	1.75	-1.25	2.19
NM_203464	*AK4*	1.74	2.43	-1.40
NM_002933	*RNASE1*	1.74	-56.26	97.90
NM_001242879	*ELP2*	1.74	2.63	-1.52
NM_001256008	*PNPLA8*	1.73	-1.80	3.11
NM_182526	*TMEM229B*	1.72	2.58	-1.50
NM_005114	*HS3ST1*	1.72	-5.24	9.01
NM_005321	*HIST1H1E*	1.71	-1.33	2.27
NM_001171157	*SIGLEC10*	1.69	7.76	-4.60
NM_006477	*RASL10A*	1.68	2.52	-1.51
NM_002041	*GABPB1*	1.67	2.45	-1.47
NM_170708	*LMNA*	1.67	-1.29	2.15
NM_015028	*TNIK*	1.66	3.02	-1.82
NM_003959	*HIP1R*	1.65	2.17	-1.31
NM_173515	*CNKSR3*	1.65	-1.73	2.86
NM_019841	*TRPV5*	1.65	-1.77	2.91
NM_001024916	*CBWD5*	1.65	-1.52	2.51

NM_001195139	*COG4*	1.64	-1.46	2.39
NM_052985	*IFT122*	1.64	-1.31	2.14
NM_001257098	*RHNO1*	1.63	-2.02	3.30
NM_005476	*GNE*	1.63	-1.54	2.51
NR_052006	*PRPF40B*	1.63	2.97	-1.82
NM_176881	*TAS2R39*	1.63	-1.47	2.39
NM_012428	*NPTN*	1.63	-1.46	2.37
NM_001271420	*TTC19*	1.63	2.34	-1.44
NM_015136	*STAB1*	1.63	-5.86	9.52
NM_004369	*COL6A3*	1.62	-4.22	6.81
NM_025139	*ARMC9*	1.62	-1.45	2.34
NM_006291	*TNFAIP2*	1.61	2.19	-1.36
NM_001253846	*GMDS*	1.60	-2.17	3.48
NM_016546	*C1RL*	1.60	5.23	-3.27
NM_001193350	*MEF2C*	1.60	-2.64	4.23
NM_006865	*LILRA3*	1.60	-1.27	2.03
NM_001552	*IGFBP4*	1.60	-1.75	2.80
NM_198545	*DRAXIN*	1.59	-1.31	2.09
NR_038441	*SLC8A1-AS1*	1.58	2.12	-1.34
NM_001207008	*MLLT4*	1.58	2.70	-1.71
NM_002030	*FPR3*	1.58	-1.37	2.16
NM_001256648	*ZNF43*	1.57	2.25	-1.43
NM_018646	*TRPV6*	1.55	-2.19	3.40
NM_001243281	*ALCAM*	1.55	-1.35	2.09
NM_032955.2	*AIF1*	1.54	-1.66	2.56
NM_001039693	*TYW5*	1.54	2.13	-1.38
NM_004417	*DUSP1*	1.54	-1.58	2.44
NM_012072	*CD93*	1.54	-3.17	4.87
NM_001025158	*CD74*	1.52	2.59	-1.70
NM_003256	*TIMP4*	1.52	-2.68	4.08
NM_001161564	*TNIK*	1.51	2.63	-1.74
NM_170774	*RASSF2*	1.51	-1.51	2.28
NM_018996	*TNRC6C*	1.51	3.00	-1.99
NM_181791	*GPR141*	1.50	3.52	-2.36
NM_000221	*KHK*	1.49	-1.52	2.27
NM_003887	*ASAP2*	1.49	2.77	-1.86
NM_001177676	*GPR68*	1.49	2.38	-1.60
NM_001271667	*DTNBP1*	1.49	2.24	-1.51
NM_001164270	*RAD51*	1.48	-1.67	2.48
NM_014737	*RASSF2*	1.48	-1.52	2.25
NM_001012302	*ANO9*	1.46	2.51	-1.72

NM_001145260	*NCOA4*	1.45	2.13	-1.47
NR_046329	*TRIM46*	1.44	2.14	-1.49
NM_182765	*HECTD2*	1.42	-1.46	2.08
NM_004951	*GPR183*	1.41	-2.42	3.42
NM_001199838	*SNX10*	1.38	3.04	-2.20
NM_001128922	*LRRC32*	1.38	8.69	-6.31
NM_002183.1	*IL3RA*	1.37	10.66	-7.75
NM_001244701	*ZFP36L1*	1.37	-1.87	2.56
NM_000250	*MPO*	1.36	2.04	-1.50
NM_001145777	*FKBP5*	1.36	2.51	-1.85
NM_015873	*VILL*	1.36	2.22	-1.63
NM_001258210	*TSKU*	1.36	3.55	-2.61
NR_040095	*LOC284801*	1.36	-1.62	2.20
NM_001127180	*MYO7A*	1.35	-2.49	3.36
NM_001128325	*SPON2*	1.35	-2.74	3.70
NM_207360	*ZC3H12D*	1.35	-1.56	2.10
NM_001195017	*CD4*	1.34	-2.97	3.99
NM_001243088	*FOXM1*	1.34	-2.51	3.36
NM_001830	*CLCN4*	1.33	2.06	-1.55
NM_001003810	*HNRNPD*	1.33	-1.71	2.27
NM_021809	*TGIF2*	1.33	2.61	-1.96
NM_002084	*GPX3*	1.32	2.77	-2.09
NM_001197115	*GCLC*	1.31	-1.78	2.34
NM_173073	*SLC35C2*	1.31	2.41	-1.84
NM_001243658	*GLRX*	1.29	3.32	-2.57
NM_174890	*ZFAND4*	1.29	2.53	-1.96
NM_001198531	*TCF7L2*	1.28	3.03	-2.36
NM_015916	*CALHM2*	1.27	-1.88	2.40
NM_020423	*SCYL3*	1.27	2.36	-1.86
NM_005606	*LGMN*	1.25	-2.87	3.58
NM_001161359	*FCHO1*	1.24	3.97	-3.22
NM_007280	*OIP5*	1.23	-1.66	2.05
NM_178040	*ERC1*	1.23	2.15	-1.75
NM_004244	*CD163*	1.22	-2.40	2.94
NM_001256689	*MELK*	1.22	-2.83	3.46
NM_134270	*SMTN*	1.22	-1.73	2.12
NM_005239	*ETS2*	1.22	2.26	-1.85
NM_001195833	*RINL*	1.22	2.10	-1.72
NM_001254738	*RND3*	1.22	-2.39	2.91
NM_005668	*ST8SIA4*	1.22	-1.81	2.20
NM_012413	*QPCT*	1.21	2.19	-1.80

NM_198235	*RNASE1*	1.21	-3.51	4.24
NM_005338	*HIP1*	1.20	2.56	-2.13
NM_001145513	*SCRN1*	1.19	-2.26	2.69
NM_001270710	*NRM*	1.19	-2.39	2.84
NM_001017535	*VDR*	1.16	3.43	-2.96
NM_032744	*ADTRP*	1.15	2.30	-1.99
NR_038316	*ITGB2-AS1*	1.13	2.17	-1.91
NM_001153552	*SCOC*	1.13	-1.85	2.10
NM_004052	*BNIP3*	1.13	-1.88	2.12
NM_015516	*TSKU*	1.12	4.28	-3.83
NM_181866	*ACOT7*	1.11	2.28	-2.05
NM_080669	*SLC46A1*	1.10	-2.59	2.85
NM_018393	*TCP11L1*	1.10	-1.85	2.03
NM_001080975	*REPS2*	1.09	-2.70	2.93
NR_073479	*IKZF5*	1.09	-3.51	3.82
NM_201613	*IKBIP*	1.08	-2.44	2.63
NM_001168376	*KIAA0319*	1.07	-2.41	2.58
NM_004184	*WARS*	1.07	2.12	-1.99
NM_001253827	*GALNT14*	1.06	-1.94	2.06
NM_006266	*RALGDS*	1.06	2.11	-1.99
NM_001145211	*SLCO2B1*	1.06	-2.21	2.33
NM_001008530	*LGMN*	1.05	-3.25	3.42
NM_172218	*SPAG1*	1.05	-1.94	2.04
NM_021914	*CFL2*	1.04	3.08	-2.94
NM_005461	*MAFB*	1.04	-2.67	2.78
NM_000698	*ALOX5*	1.03	4.96	-4.81
NM_004051	*BDH1*	1.03	2.02	-1.96
NM_001142675	*CHID1*	1.03	-2.17	2.23
NM_138399	*TMEM44*	1.02	-2.08	2.12
NR_027473	*KDM2A*	1.01	2.39	-2.36
NM_002566	*P2RY11*	1.01	-2.27	2.30
NR_038230	*LOC284080*	1.01	-2.01	2.03
NM_001193348	*MEF2C*	1.01	-2.26	2.28
NM_203416	*CD163*	1.01	-3.22	3.24
NM_152889	*CHST13*	1.01	-2.35	2.37
NM_001033723	*ZNF704*	1.00	-2.06	2.06
NM_198234	*RNASE1*	1.00	-2.71	2.71
NM_198232	*RNASE1*	1.00	-2.72	2.72
NM_001845	*COL4A1*	1.00	-2.77	2.77
NM_001207066	*CXADR*	1.00	-2.97	2.97
NM_001083963	*TDRKH*	-1.01	-2.04	2.03

NM_007173	*PRSS23*	-1.01	-2.96	2.92
NM_001017536	*VDR*	-1.02	2.32	-2.36
NM_170587	*RGS20*	-1.02	-2.28	2.23
NM_005009	*NME4*	-1.02	-2.07	2.02
NR_038304	*LOC100505702*	-1.03	3.49	-3.59
NM_182755	*ZNF438*	-1.04	2.47	-2.56
NM_003048	*SLC9A2*	-1.04	-3.35	3.21
NM_001128301	*LYRM1*	-1.05	2.05	-2.14
NM_003808	*TNFSF13*	-1.06	2.11	-2.24
NM_001002269	*EXOSC3*	-1.06	-2.29	2.16
NM_004506	*HSF2*	-1.06	-2.67	2.51
NM_002937	*RNASE4*	-1.07	-2.83	2.66
NM_199362	*TPD52L2*	-1.07	-2.14	2.01
NR_033963	*FLJ44511*	-1.07	1.89	-2.02
NM_004454	*ETV5*	-1.07	-2.74	2.56
NM_178496	*MB21D2*	-1.08	2.12	-2.28
NM_018841	*GNG12*	-1.08	-2.26	2.10
NM_014238	*KSR1*	-1.09	1.97	-2.14
NM_181482	*LDLRAD4*	-1.09	-2.86	2.63
NM_005252	*FOS*	-1.10	-2.12	1.93
NM_007256	*SLCO2B1*	-1.10	-2.06	1.88
NM_012338	*TSPAN12*	-1.10	-2.29	2.09
NM_181093	*SCYL3*	-1.11	-2.80	2.53
NM_001040441	*ZBTB8A*	-1.11	1.90	-2.11
NM_080706	*TRPV1*	-1.12	1.99	-2.24
NM_001500	*GMDS*	-1.12	2.71	-3.05
NM_004715	*CTDP1*	-1.13	2.55	-2.87
NM_005544	*IRS1*	-1.13	-2.20	1.95
NM_001338	*CXADR*	-1.13	-5.11	4.53
NM_017449	*EPHB2*	-1.13	-2.15	1.90
NM_001171820	*PPARD*	-1.13	1.97	-2.23
NM_001143682	*CALCOCO1*	-1.14	1.82	-2.08
NM_002348	*LY9*	-1.16	-2.47	2.14
NM_021070	*LTBP3*	-1.16	1.95	-2.27
NM_005501	*ITGA3*	-1.17	-4.27	3.65
NM_152219	*GJD3*	-1.17	2.02	-2.36
NM_001256154	*ALOX5*	-1.18	4.38	-5.15
NM_004925	*AQP3*	-1.18	2.27	-2.67
NR_047676	*TARS*	-1.19	2.22	-2.64
NR_073111	*SERPINB1*	-1.19	1.94	-2.31
NM_139055	*ADAMTS15*	-1.20	-3.48	2.90

NM_001127444	*CD36*	-1.20	-3.33	2.77
NM_138809	*CMBL*	-1.20	-2.09	1.74
NM_001257134	*COX14*	-1.20	-2.34	1.94
NM_032868	*MPND*	-1.21	1.74	-2.09
NM_024769	*CLMP*	-1.21	-5.34	4.41
NM_001271840	*IKZF5*	-1.21	2.83	-3.44
NM_001177984	*SCN8A*	-1.23	-2.23	1.81
NM_001256238	*PGAP2*	-1.23	-2.99	2.43
NM_005962	*MXI1*	-1.24	-3.34	2.69
NM_018248	*NEIL3*	-1.25	-2.06	1.65
NM_001945	*HBEGF*	-1.25	2.70	-3.37
NM_005876	*SPEG*	-1.25	-2.59	2.07
NM_145071	*CISH*	-1.26	3.12	-3.93
NM_005168	*RND3*	-1.26	-5.51	4.37
NM_015600	*ABHD12*	-1.26	-2.03	1.61
NM_016025	*METTL9*	-1.26	1.67	-2.11
NM_138554	*TLR4*	-1.27	-2.26	1.78
NM_207103	*SCIMP*	-1.27	2.03	-2.59
NM_000351	*STS*	-1.28	1.73	-2.21
NM_016467	*ORMDL1*	-1.28	1.73	-2.21
NM_003947	*KALRN*	-1.29	-2.66	2.07
NM_019032	*ADAMTSL4*	-1.29	3.45	-4.47
NM_000260	*MYO7A*	-1.30	-2.72	2.10
NM_001080529	*WIPF3*	-1.30	-2.21	1.70
NM_197941	*ADAMTS6*	-1.30	-3.08	2.37
NM_001109891	*MAPK3*	-1.31	-3.00	2.29
NM_001243926	*MAPKAPK3*	-1.31	2.79	-3.66
NM_004274	*AKAP6*	-1.31	-4.78	3.64
NM_032667	*BSCL2*	-1.32	-2.01	1.52
NM_173065	*IFNLR1*	-1.32	-2.39	1.81
NM_017637	*BNC2*	-1.32	-3.39	2.56
NM_000789	*ACE*	-1.33	3.22	-4.29
NM_001243540	*LOC100653515*	-1.33	1.69	-2.25
NM_018071	*ARHGEF40*	-1.34	-2.07	1.55
NM_002311	*LIG3*	-1.34	2.04	-2.74
NM_001010863	*C10orf128*	-1.34	2.07	-2.79
NM_001242490	*FAM156A*	-1.36	2.54	-3.44
NM_175839	*SMOX*	-1.37	4.56	-6.22
NM_014922	*NLRP1*	-1.38	1.82	-2.51
NM_003890	*FCGBP*	-1.38	-19.15	13.89
NM_014055	*IFT81*	-1.38	-2.32	1.68

NM_199452	*ZNF365*	-1.38	2.23	-3.07
NM_002424	*MMP8*	-1.38	-4.66	3.37
NM_001011655	*TMEM44*	-1.39	-2.19	1.58
NM_014945	*ABLIM3*	-1.39	-4.05	2.92
NM_003423	*ZNF43*	-1.39	-2.64	1.89
NM_013276	*SHPK*	-1.42	1.53	-2.17
NM_001184768	*ARMCX6*	-1.42	-2.32	1.63
NM_001242412	*AHRR*	-1.42	-3.78	2.66
NM_001001548	*CD36*	-1.43	-3.65	2.56
NM_030891	*LRRC3*	-1.43	-2.27	1.58
NR_034082	*LOC100130950*	-1.43	2.09	-2.99
NM_001242533	*MFSD11*	-1.44	1.65	-2.38
NM_006329	*FBLN5*	-1.44	-5.80	4.03
NM_052883	*TXNRD3*	-1.44	-2.79	1.93
NM_207370	*GPR153*	-1.44	1.97	-2.85
NM_139249	*MS4A6E*	-1.45	-2.76	1.91
NM_001202403	*ASB6*	-1.46	1.53	-2.24
NM_001190720	*IKBKB*	-1.46	1.44	-2.11
NM_002204	*ITGA3*	-1.47	-3.85	2.62
NM_002445	*MSR1*	-1.47	-2.77	1.88
NM_000376	*VDR*	-1.49	2.03	-3.01
NM_001252660	*EPB41L2*	-1.49	-3.52	2.36
NM_002438.1	*MRC1*	-1.49	2.02	-3.01
NM_002438	*MRC1*	-1.50	2.05	-3.06
NM_001146218	*WRB*	-1.50	-3.21	2.14
NM_001098784	*FAM89B*	-1.51	1.54	-2.32
NM_000313	*PROS1*	-1.51	1.59	-2.41
NR_036493	*PEX19*	-1.52	-2.48	1.63
NM_001006945	*LRIF1*	-1.53	-2.30	1.51
NM_004442	*EPHB2*	-1.53	-2.83	1.85
NM_001271368	*PTGS1*	-1.54	1.46	-2.25
NM_172089	*TNFSF12-TNFSF13*	-1.54	-2.13	1.39
NM_016289	*CAB39*	-1.54	-2.28	1.48
NM_001166305	*TMEM44*	-1.54	1.45	-2.23
NM_004613	*TGM2*	-1.54	1.56	-2.40
NM_025008	*ADAMTSL4*	-1.54	1.69	-2.61
NM_001040456	*RHBDD2*	-1.55	1.73	-2.69
NM_005688	*ABCC5*	-1.55	-2.07	1.33
NM_001346	*DGKG*	-1.56	-3.19	2.05
NM_001461	*FMO5*	-1.57	1.44	-2.26
NM_001160125	*KLF6*	-1.59	-3.15	1.98

NM_001127628	*FBP1*	-1.59	1.79	-2.85
NR_037945	*STX16-NPEPL1*	-1.59	1.65	-2.64
NM_001040457	*RHBDD2*	-1.61	4.13	-6.65
NR_047594	*CENPJ*	-1.62	2.24	-3.62
NM_001143965	*TBC1D7*	-1.62	2.07	-3.35
NR_046349	*PPWD1*	-1.62	-3.63	2.24
NM_183240	*TMEM37*	-1.62	-5.04	3.10
NM_001976	*ENO3*	-1.63	3.09	-5.03
NR_038241	*VEZT*	-1.64	-2.78	1.69
NR_037705	*ZNF778*	-1.64	2.32	-3.81
NR_045720	*MAN1B1*	-1.64	-2.17	1.32
NR_038966	*LOC100506585*	-1.66	1.35	-2.23
NM_203446	*SYNJ1*	-1.66	1.21	-2.02
NM_001134707	*SARDH*	-1.67	1.38	-2.31
NM_001198619	*MAP7*	-1.68	2.56	-4.29
NM_024761	*MOB3B*	-1.68	1.85	-3.10
NM_001145642	*KIAA0226*	-1.68	1.25	-2.10
NM_005309	*GPT*	-1.70	1.34	-2.27
NR_047530	*SIGLEC17P*	-1.70	1.18	-2.01
NM_001012968	*SPIN4*	-1.70	1.62	-2.76
NM_001045556	*SLA*	-1.70	1.18	-2.01
NM_003039	*SLC2A5*	-1.71	-2.19	1.28
NM_005781	*TNK2*	-1.72	1.41	-2.43
NM_030769	*NPL*	-1.72	-2.30	1.34
NM_000676	*ADORA2B*	-1.74	-2.17	1.25
NM_001083314	*CHMP1A*	-1.74	-2.05	1.18
NR_036641	*PDGFC*	-1.75	2.16	-3.78
NM_016084	*RASD1*	-1.76	1.16	-2.03
NM_006343	*MERTK*	-1.76	-4.43	2.52
NR_038080	*LINC00482*	-1.76	1.54	-2.72
NM_001127443	*CD36*	-1.77	-4.25	2.40
NM_018330	*KIAA1598*	-1.79	-3.54	1.98
NM_020431	*TMEM63C*	-1.79	1.39	-2.49
NM_173505	*ANKRD29*	-1.81	1.76	-3.18
NM_005559	*LAMA1*	-1.81	-3.03	1.68
NM_203505	*G3BP2*	-1.81	1.31	-2.37
NM_001112	*ADARB1*	-1.82	-2.36	1.29
NM_003895	*SYNJ1*	-1.82	1.39	-2.52
NM_001505	*GPER*	-1.83	1.32	-2.41
NM_006843	*SDS*	-1.83	-3.58	1.95
NM_207123	*GAB1*	-1.83	-2.51	1.37

NM_001243248	*NPRL3*	-1.84	1.28	-2.36
NM_000072	*CD36*	-1.84	-4.83	2.62
NM_052851	*STARD13*	-1.85	-4.66	2.52
NM_018374	*TMEM106B*	-1.85	1.26	-2.32
NM_005615	*RNASE6*	-1.87	-2.90	1.55
NM_001172569	*MYD88*	-1.87	1.09	-2.04
NM_030636	*EEPD1*	-1.87	-2.44	1.31
NM_080705	*TRPV1*	-1.87	1.38	-2.59
NM_000787	*DBH*	-1.88	1.68	-3.16
NM_001102610	*TUBGCP5*	-1.89	-2.20	1.17
NM_003174	*SVIL*	-1.89	1.26	-2.38
NM_153640	*PANK2*	-1.90	-2.50	1.32
NM_001270965	*PSD*	-1.90	1.41	-2.69
NM_001010938	*TNK2*	-1.94	1.45	-2.81
NR_026745	*COG6*	-1.96	-2.16	1.10
NM_004447	*EPS8*	-1.98	-2.20	1.11
NM_004235	*KLF4*	-1.99	1.88	-3.74
NM_052972	*LRG1*	-1.99	1.56	-3.12
NM_052847	*GNG7*	-2.00	1.14	-2.28
NM_001256574	*ENC1*	-2.00	-3.32	1.66
NM_001159920	*FLT1*	-2.00	-1.14	-1.76
NM_001164638	*ENDOV*	-2.00	-1.74	-1.15
NM_001145829	*TNNI2*	-2.01	-2.23	1.11
NM_001033053	*NLRP1*	-2.01	1.99	-4.00
NM_001261451	*UCK1*	-2.01	-1.13	-1.77
NM_000180	*GUCY2D*	-2.02	-1.06	-1.90
NM_001024660	*KALRN*	-2.02	-2.42	1.20
NM_020153	*IFT46*	-2.02	-2.74	1.35
NM_016060	*MED31*	-2.02	-1.30	-1.55
NM_001173533	*DGCR2*	-2.03	-1.75	-1.16
NM_024661	*CCDC51*	-2.03	-2.62	1.29
NR_003336	*SNORD116-22*	-2.04	-2.09	1.03
NR_049755	*RPP14*	-2.04	1.56	-3.19
NR_023344	*RNU6ATAC*	-2.04	-2.05	1.00
NM_001142595	*P4HA1*	-2.05	-1.31	-1.56
NM_007037	*ADAMTS8*	-2.05	-4.84	2.36
NM_005980	*S100P*	-2.05	1.16	-2.39
NM_001204082	*MINOS1*	-2.06	1.00	-2.06
NM_003644	*GAS7*	-2.06	-2.95	1.43
NM_015122	*FCHO1*	-2.06	-2.41	1.17
NM_203342	*EPB41*	-2.07	1.18	-2.45

NM_178500	*PHOSPHO1*	-2.07	-1.62	-1.28
NM_002612	*PDK4*	-2.08	-3.89	1.87
NM_001003694	*BRPF1*	-2.08	-1.44	-1.44
NM_174918	*C19orf59*	-2.08	4.70	-9.76
NM_198282	*TMEM173*	-2.08	1.36	-2.83
NM_001031804	*MAF*	-2.09	-2.04	-1.02
NR_003013	*SCARNA16*	-2.09	-1.48	-1.41
NM_007261	*CD300A*	-2.09	-1.97	-1.06
NM_152536	*FGD5*	-2.09	1.02	-2.13
NM_001252152	*SLC39A9*	-2.10	1.41	-2.94
NM_153436	*ODF2*	-2.10	-2.42	1.15
NM_130439	*MXI1*	-2.10	-1.63	-1.29
NM_001258447	*TOP1MT*	-2.10	-1.15	-1.82
NM_001001547	*CD36*	-2.10	-6.92	3.29
NM_001198690	*PPAN-P2RY11*	-2.10	-2.29	1.09
NM_005657	*TP53BP1*	-2.10	-3.36	1.60
NM_004807	*HS6ST1*	-2.13	-1.34	-1.59
NM_022746	*1-Mar*	-2.13	-1.68	-1.26
NM_032506	*KIAA1841*	-2.13	-2.54	1.19
NM_139349	*BIN1*	-2.14	-1.04	-2.05
NR_003015	*SNORA53*	-2.14	-2.79	1.31
NM_001243168	*PTCRA*	-2.14	1.03	-2.21
NR_026591	*RAD1*	-2.14	1.39	-2.97
NR_049745.1	*HYDIN2*	-2.14	-1.40	-1.53
NM_001042479	*GEMIN8*	-2.14	-3.44	1.60
NM_001193644	*PEX19*	-2.15	-1.03	-2.08
NM_198310	*TTC8*	-2.15	-2.93	1.37
NM_000530	*MPZ*	-2.15	-1.80	-1.19
NR_038217	*LOC154092*	-2.15	-1.37	-1.57
NM_001257317	*DCSTAMP*	-2.15	-1.20	-1.80
NR_003025	*SNORA59A*	-2.16	-3.02	1.40
NR_003022	*SNORA59B*	-2.16	-3.02	1.40
NM_001099679	*TRIM32*	-2.16	-2.46	1.14
NM_000573	*CR1*	-2.17	1.08	-2.34
NM_152443	*RDH12*	-2.17	1.17	-2.54
NM_001077241	*SLC25A45*	-2.18	-2.52	1.16
NR_003317	*SNORD116-2*	-2.18	-2.02	-1.08
NM_152720	*NEK3*	-2.20	-1.03	-2.14
NR_002971	*SNORA38*	-2.20	-2.36	1.07
NM_033004	*NLRP1*	-2.20	1.80	-3.95
NR_002325	*SNORA6*	-2.22	-2.04	-1.09

NM_001142315	*LMO2*	-2.23	-1.00	-2.22
NM_002873	*RAD17*	-2.23	-1.80	-1.24
NM_024781	*CCDC102B*	-2.24	-1.01	-2.22
NM_145759	*TRAF5*	-2.25	-2.19	-1.03
NM_001142575	*IMPDH1*	-2.26	-2.06	-1.09
NM_001098201	*GPER*	-2.27	-1.22	-1.86
NM_001014811	*ME3*	-2.28	-5.12	2.25
NM_001270699	*ARHGAP12*	-2.28	-1.61	-1.41
NM_153690	*FAM43A*	-2.28	-1.55	-1.46
NM_001143999	*SEC14L1*	-2.29	-1.54	-1.49
NM_004753	*DHRS3*	-2.29	-1.33	-1.72
NM_001098531	*RAPGEF3*	-2.30	-1.89	-1.22
NM_005147	*DNAJA3*	-2.30	-1.65	-1.39
NM_052904	*KLHL32*	-2.30	-1.98	-1.16
NM_016562	*TLR7*	-2.31	-2.59	1.13
NM_001143841	*TMEM106C*	-2.31	-3.34	1.44
NM_004338	*LDLRAD4*	-2.31	1.11	-2.56
NM_001040667	*HSF4*	-2.32	-1.36	-1.70
NM_001198568	*ADCY4*	-2.32	-1.52	-1.52
NR_033579.1	*LOC440297*	-2.32	-2.51	1.08
NM_001080	*ALDH5A1*	-2.35	-1.26	-1.87
NM_021116	*ADCY1*	-2.35	-8.01	3.41
NM_001178008	*CBS*	-2.36	1.27	-2.99
NM_001242394	*SYTL3*	-2.36	1.16	-2.73
NR_002964	*SNORA28*	-2.36	-2.05	-1.15
NM_006484	*DYRK1B*	-2.37	-1.62	-1.46
NM_031220	*PITPNM3*	-2.37	1.17	-2.77
NM_001080956	*PLAGL1*	-2.38	-5.57	2.35
NM_001256496	*MAN2C1*	-2.39	-1.27	-1.87
NM_175834	*KRT79*	-2.39	-1.61	-1.49
NM_001142578	*ZNF780A*	-2.39	-3.63	1.52
NM_183065	*TMEM107*	-2.39	-1.88	-1.27
NM_181716	*CENPV*	-2.42	-1.84	-1.32
NM_001100429	*RAP1GDS1*	-2.42	-3.22	1.33
NM_001270623	*SLC16A7*	-2.42	-2.66	1.10
NM_021245	*MYOZ1*	-2.43	-1.36	-1.79
NR_002978	*SNORA46*	-2.43	-1.99	-1.22
NM_145173	*DIRAS1*	-2.44	-1.94	-1.26
NM_203370	*FAM212A*	-2.45	-1.26	-1.95
NR_024195	*TOM1*	-2.45	-2.59	1.06
NM_001129998	*CLEC12B*	-2.45	1.06	-2.59

NM_052963	*TOP1MT*	-2.46	-1.83	-1.34
NM_001135051	*FAM160B1*	-2.46	-1.80	-1.37
NM_001185158	*IL24*	-2.46	-2.63	1.07
NM_001199723	*CRABP2*	-2.46	-1.64	-1.50
NR_037803	*BACE1-AS*	-2.47	-1.63	-1.51
NM_001244	*TNFSF8*	-2.47	-4.80	1.95
NM_032354	*TMEM107*	-2.47	-1.93	-1.28
NM_001135812	*FAM60A*	-2.47	-2.41	-1.02
NM_001166349	*SLC26A11*	-2.47	1.01	-2.50
NM_001160230	*ADARB1*	-2.48	-3.03	1.22
NM_001015056	*RTKN*	-2.50	-1.96	-1.28
NM_213618	*ST5*	-2.51	-1.34	-1.87
NM_173626	*SLC26A11*	-2.52	-1.57	-1.61
NM_033110	*GGACT*	-2.52	-1.66	-1.52
NM_152463	*EME1*	-2.54	-2.05	-1.24
NM_173681	*ATG9B*	-2.55	-1.29	-1.97
NR_028057.1	*PLCXD1*	-2.55	-1.20	-2.13
NM_152996	*ST6GALNAC3*	-2.55	-1.12	-2.28
NR_003334	*SNORD116-20*	-2.56	-2.28	-1.12
NM_173064	*IFNLR1*	-2.56	-2.95	1.15
NM_025080	*ASRGL1*	-2.57	1.11	-2.85
NR_024621	*C11orf21*	-2.58	-1.43	-1.81
NM_001127213	*LY6E*	-2.60	-1.53	-1.70
NR_047529	*SIGLEC17P*	-2.61	-2.26	-1.15
NM_052820	*CORO2A*	-2.61	-2.29	-1.14
NM_033006	*NLRP1*	-2.62	1.71	-4.49
NM_001185099	*CD22*	-2.62	-1.10	-2.39
NM_001098202	*HIC1*	-2.63	1.92	-5.03
NM_001242948	*EPDR1*	-2.63	-3.45	1.31
NR_003026	*SNORA1*	-2.63	-2.30	-1.15
NM_019089	*HES2*	-2.64	1.27	-3.35
NM_198156	*VHL*	-2.64	-1.91	-1.38
NR_002955	*SNORA14A*	-2.64	-1.29	-2.05
NM_002019	*FLT1*	-2.65	-1.28	-2.07
NM_001267708	*ZNF706*	-2.67	-1.92	-1.39
NM_002346	*LY6E*	-2.67	-1.80	-1.48
NM_170740	*ALDH5A1*	-2.67	-1.83	-1.46
NM_001080824	*TRABD2A*	-2.68	1.38	-3.71
NM_001102605	*IMPDH1*	-2.68	-1.90	-1.41
NR_038303	*LOC100505702*	-2.69	-1.14	-2.36
NM_001098844.1	*TMEM236*	-2.69	2.66	-7.15

NM_021913	*AXL*	-2.73	1.08	-2.94
NM_001270402	*KCNE1*	-2.74	-1.05	-2.60
NR_003709	*SNORA11B*	-2.74	-1.79	-1.53
NM_001172663	*RAB40C*	-2.74	1.00	-2.74
NR_003339	*SNORD116-25*	-2.74	-2.19	-1.25
NR_046315	*BLOC1S2*	-2.76	-1.08	-2.56
NM_133168	*OSCAR*	-2.76	-3.29	1.19
NM_144601	*CMTM3*	-2.77	-2.06	-1.34
NM_031293	*PMFBP1*	-2.77	-4.15	1.50
NM_006105	*RAPGEF3*	-2.77	-2.81	1.01
NM_000860	*HPGD*	-2.81	-1.83	-1.54
NM_018719	*CDCA7L*	-2.81	-1.47	-1.92
NM_001135602	*GLB1*	-2.83	-1.59	-1.77
NM_001199620	*NCOA7*	-2.85	-3.25	1.14
NM_003282	*TNNI2*	-2.86	-3.09	1.08
NM_001766	*CD1D*	-2.86	-1.53	-1.87
NM_001079526	*IKZF2*	-2.86	-1.32	-2.17
NM_001193431	*PTPN22*	-2.87	-1.89	-1.52
NR_004862	*TYW5*	-2.89	-3.63	1.26
NM_080704	*TRPV1*	-2.90	1.67	-4.86
NM_207128	*PAOX*	-2.91	1.39	-4.05
NM_001001925	*MTUS1*	-2.94	-3.80	1.29
NM_020975	*RET*	-2.94	1.09	-3.19
NM_001207048	*BACE1*	-2.95	-1.31	-2.25
NR_073072.4	*NRM*	-2.96	-2.17	-1.37
NM_001243013	*ABCB9*	-2.96	-2.61	-1.13
NM_001168478	*ARMCX5*	-2.98	-2.44	-1.22
NM_001267042	*ARMC8*	-2.99	-3.09	1.03
NR_033191	*PPP2R2D*	-2.99	-1.93	-1.55
NM_005582	*CD180*	-2.99	-4.92	1.64
NM_001168278	*WWTR1*	-3.00	-1.55	-1.94
NM_001202412	*ZNF559*	-3.01	-2.76	-1.09
NM_001199480	*ZNF193*	-3.02	-3.40	1.13
NR_003105	*ZWILCH*	-3.07	-1.40	-2.19
NM_016150	*ASB2*	-3.10	-1.67	-1.85
NM_001168280	*WWTR1*	-3.11	-2.45	-1.27
NM_001143888	*BSDC1*	-3.13	-2.18	-1.44
NM_006744	*RBP4*	-3.13	-1.66	-1.89
NR_026759	*FAM95B1*	-3.15	-2.98	-1.06
NM_033102	*SLC45A3*	-3.16	-2.87	-1.10
NR_026759.1	*FAM95B1*	-3.20	-1.36	-2.35

NM_006094	*DLC1*	-3.20	1.17	-3.75
NM_005300	*GPR34*	-3.21	-3.65	1.14
NM_001164730	*REEP1*	-3.25	-1.35	-2.40
NR_046401	*ASIC3*	-3.26	1.09	-3.54
NM_001098844	*TMEM236*	-3.28	2.19	-7.18
NM_003888	*ALDH1A2*	-3.28	1.24	-4.06
NM_022822	*KLC2*	-3.30	-1.60	-2.07
NM_001242384	*SYTL3*	-3.32	-1.37	-2.43
NM_015210	*SOGA2*	-3.32	-1.82	-1.82
NM_014298	*QPRT*	-3.32	-2.36	-1.41
NR_026864	*PRSS30P*	-3.37	1.43	-4.84
NM_001102592	*HENMT1*	-3.42	-1.53	-2.23
NM_001442	*FABP4*	-3.44	-11.16	3.24
NM_001256430	*STON2*	-3.45	1.36	-4.69
NM_001166270	*HAUS4*	-3.45	-2.28	-1.51
NM_001135825	*RNF185*	-3.45	-2.02	-1.71
NM_001144962.3	*NFKBIL1*	-3.46	-4.03	1.16
NM_001629	*ALOX5AP*	-3.50	1.60	-5.61
NM_001256599	*TRIM46*	-3.60	-1.55	-2.32
NM_001172633	*OLR1*	-3.62	1.62	-5.84
NM_021154	*PSAT1*	-3.62	-1.15	-3.14
NM_130782	*RGS18*	-3.62	-4.78	1.32
NR_036527	*LOC100499484*	-3.64	-1.65	-2.20
NM_001193308	*SYTL1*	-3.67	-1.51	-2.43
NM_170697	*ALDH1A2*	-3.68	1.94	-7.15
NM_020415	*RETN*	-3.70	1.04	-3.83
NM_052924	*RHPN1*	-3.70	-2.16	-1.71
NM_018371	*CSGALNACT1*	-3.72	-1.64	-2.26
NM_199280	*FAM179A*	-3.74	-2.78	-1.34
NM_001271146	*ST3GAL6*	-3.75	-1.53	-2.45
NR_045107	*VWA9*	-3.75	-2.20	-1.70
NM_001197140	*DBNDD2*	-3.76	-1.44	-2.60
NM_003149	*STAC*	-3.90	5.37	-20.95
NM_006680	*ME3*	-3.90	-3.87	-1.01
NM_003656	*CAMK1*	-3.92	-1.81	-2.16
NM_004864	*GDF15*	-3.94	-2.18	-1.81
NM_058248	*DNASE2B*	-3.97	-2.95	-1.35
NM_001145657	*RAP1GAP*	-3.98	1.17	-4.64
NM_001168388	*CITED2*	-3.99	-5.31	1.33
NR_003009	*SCARNA8*	-4.01	-2.86	-1.40
NM_001134415	*TBCCD1*	-4.05	-2.51	-1.61

NM_001244364	*ARHGEF28*	-4.05	1.20	-4.88
NM_001136268	*DAZAP2*	-4.11	-1.64	-2.50
NM_001098824	*TMEM91*	-4.20	-1.89	-2.23
NM_020686	*ABAT*	-4.25	-2.40	-1.77
NM_020971	*SPTBN4*	-4.25	-2.51	-1.69
NM_002885	*RAP1GAP*	-4.26	-1.13	-3.77
NM_033104	*STON2*	-4.29	1.18	-5.05
NM_001040260	*DCLK2*	-4.32	-2.50	-1.73
NM_018070	*SSBP3*	-4.35	-1.72	-2.54
NM_153266	*TMEM151A*	-4.39	-1.59	-2.75
NM_001129727	*PLEKHG4*	-4.42	-2.94	-1.51
NM_001202429	*ASB2*	-4.47	-3.30	-1.36
NM_022349	*MS4A6A*	-4.48	-7.10	1.58
NM_001081004	*COMMD5*	-4.57	-2.60	-1.76
NM_001145658	*RAP1GAP*	-4.59	-1.27	-3.60
NR_045573	*ISYNA1*	-4.63	-1.26	-3.69
NM_005410	*SEPP1*	-4.64	-44.90	9.68
NM_001816	*CEACAM8*	-4.64	-1.05	-4.44
NM_015985	*ANGPT4*	-4.68	-3.33	-1.41
NM_002991	*CCL24*	-4.76	7.85	-37.38
NM_177477	*LYNX1*	-5.01	-1.15	-4.34
NM_001029883	*C2orf71*	-5.45	-2.19	-2.49
NM_018013	*SOBP*	-5.50	1.21	-6.63
NM_003236	*TGFA*	-5.82	-1.02	-5.72
NM_001270895	*TRAPPC3*	-5.93	-2.06	-2.87
NM_006497	*HIC1*	-6.89	-1.18	-5.85
NM_001099691	*TGFA*	-7.49	-1.51	-4.95
NM_001143919	*LTB4R*	-7.76	-1.83	-4.24
NM_001764	*CD1B*	-11.15	-1.71	-6.51
NM_001207019	*FCER2*	-16.79	-1.56	-10.79

Supplementary Table 2. Select transcripts significantly differentially expressed between M-MDM and GM-MDM

RefSeq transcript ID	Gene symbol	Entrez Gene Name	FC (vs. GM-MDM)
NM_00293	*RNASE1*	Ribonuclease, RNase A family, 1	97.90
NM_002160	*TNC*	Tenascin C	20.32
NM_003890	*FCGBP*	Fc fragment of IgG binding protein	13.89
NM_001142343	*CMKLR1*	Chemerin chemokine-like receptor 1	11.22
NM_006840	*LILRB5*	Leukocyte immunoglobulin like receptor B	10.97
NM_005410	*SEPP1*	Selenoprotein P, plasma, 1	9.68
NM_015136	*STAB1*	Stabilin 1	9.52
NM_014479	*ADAMDEC1*	ADAM-like, decysin 1	9.03
NM_002982	*CCL2*	Chemokine (C-C motif) ligand 2	8.36
NM_012072	*CD93*	CD93 molecule	4.87
NM_015149	*RGL1*	Ral guanine nucleotide dissociation stimulator-like 1	4.76
NM_006329	*FBLN5*	Fibulin 5	4.03
NM_005118	*TNFSF15*	Tumor necrosis factor superfamily member 15	3.84
NM_004994	*MMP9*	Matrix metallopeptidase 9	3.73
NM_005606	*LGMN*	Legumain	3.58
NM_004530	*MMP2*	Matrix metallopeptidase 2	3.50
NM_001008530	*LGMN*	Legumain	3.42
NM_002424	*MMP8*	Matrix metallopeptidase 8	3.37
NM_001001547	*CD36*	CD36 molecule	3.29
NM_203416	*CD163*	CD163 molecule	3.24
NM_004244	*CD163*	CD163 molecule	2.94
NM_001127444	*CD36*	CD36 molecule	2.77
NM_000072	*CD36*	CD36 molecule	2.62
NM_001001548	*CD36*	CD36 molecule	2.56
NM_001127443	*CD36*	CD36 molecule	2.40
NM_002985	*CCL5*	Chemokine (C-C motif) ligand 5	2.31
NM_020530	*OSM*	Oncostatin M	2.26
NM_004052	*BNIP3*	BCL2/adenovirus E1B 19kDa interacting protein 3	2.12

NM_002019	*FLT1*	FMS-related tyrosine kinase 1	-2.07
NM_002122	*HLA-DQA1*	Major histocompatibility complex, class II, DQα1	-2.24
NM_003808	*TNFSF13*	Tumor necrosis factor superfamily member 13	-2.24
NM_001955	*EDN1*	Endothelin 1	-2.5
NM_002438.1	*MRC1*	Mannose receptor, C type 1	-3.01
NM_002438	*MRC1*	Mannose receptor, C type 1	-3.06
NM_001145658	*RAP1GAP*	RAP1 GTPase activating protein	-3.60
NM_002885	*RAP1GAP*	RAP1 GTPase activating protein	-3.77
NM_001145657	*RAP1GAP*	RAP1 GTPase activating protein	-4.64
NM_001099691	*TGFA*	Transforming growth factor α	-4.95
NM_003236	*TGFA*	Transforming growth factor α	-5.72
NM_002183	*IL3RA*	Interleukin 3 receptor subunit α	-6.20
NM_001764	*CD1B*	CD1b molecule	-6.51
NM_002183.1	*IL3RA*	Interleukin 3 receptor subunit α	-7.75
NM_001207019	*FCER2*	Fc fragment of IgE receptor II	-10.79
NM_003149	*STAC*	SH3 and cysteine rich domain	-20.95
NM_002991	*CCL24*	Chemokine (C-C motif) ligand 24	-37.38

FC, fold-change

Supplementary Table 3. Transcripts that are uniquely and significantly differentially expressed in Mob-MDM compared to both GM-MDM and M-MDM

		Fold Change		
**RefSeq transcript ID**	**Symbol**	**Mob-MDM vs. GM-MDM**	**Mob-MDM vs. M-MDM**	**M-MDM vs. GM-MDM**
**Chemokines and cytokines**				
NM_000576	*IL1B*	20.54	13.04	1.58
NM_000584	*IL8*	11.89	22.67	-1.91
NM_002984	*CCL4*	10.37	7.04	1.47
NM_002988	*CCL18*	9.00	17.35	-1.93
NM_207007.1	*CCL4L2*	8.17	5.48	1.49
NM_001001435.1	*CCL4L1*	8.17	5.48	1.49
NM_002416	*CXCL9*	7.21	6.82	1.06
NM_207007	*CCL4L2*	6.07	4.61	1.32
NM_001001435	*CCL4L1*	6.07	4.61	1.32
NM_000594.5	*TNF*	5.67	3.65	1.55
NM_001511	*CXCL1*	5.15	3.72	1.38
NM_021006	*CCL3L1*	5.04	5.37	-1.07
NM_005746	*NAMPT*	3.75	2.02	1.86
NM_005755	*EBI3*	3.27	3.27	1.00
NM_002983	*CCL3*	2.47	2.87	-1.16
NM_002341.4	*LTB*	2.28	2.53	-1.11
NM_005064	*CCL23*	2.26	2.69	-1.19
NR_046035	*CXCL1*	2.25	2.07	1.09
NM_001130046	*CCL20*	2.08	2.08	1.00
NM_001185158	*IL24*	-2.46	-2.63	1.07
NM_144601	*CMTM3*	-2.77	-2.06	-1.34
**G-protein coupled receptors**				
NM_001838	*CCR7*	7.42	5.00	1.48
NM_001142345	*CMKLR1*	4.55	4.39	1.04
NM_000675	*ADORA2A*	4.23	4.15	1.02
NM_002029	*FPR1*	3.18	2.54	1.25
NM_003507	*FZD7*	2.67	3.12	-1.17
NM_001992	*F2R*	2.32	2.32	1.00
NM_005300	*GPR34*	-3.21	-3.65	1.14
**Growth Factors**				
NM_000266	*NDP*	5.01	3.93	1.27
NM_004864	*GDF15*	-3.94	-2.18	-1.81

NM_015985	*ANGPT4*	-4.68	-3.33	-1.41
**Ion Channels**				
NM_153259	*MCOLN2*	9.28	5.81	1.60
NM_001135775	*CACFD1*	3.85	4.81	-1.25
NM_002224	*ITPR3*	2.27	2.12	1.07
**Kinases**				
NM_000215	*JAK3*	5.26	6.37	-1.21
NM_001570	*IRAK2*	3.78	2.20	1.72
NR_027767	*TNIK*	2.37	2.17	1.09
NM_001161560	*TNIK*	2.27	2.54	-1.12
NM_016441	*CRIM1*	2.17	3.74	-1.73
NM_001005353	*AK4*	2.05	2.44	-1.19
NM_001024660	*KALRN*	-2.02	-2.42	1.20
**Peptidases**				
NM_201442	*C1S*	31.38	40.36	-1.29
NM_004131	*GZMB*	10.34	10.34	1.00
NM_033274	*ADAM19*	8.14	8.34	-1.02
NM_000064	*C3*	7.29	5.09	1.43
NM_004995	*MMP14*	6.87	4.50	1.53
NM_001145271	*ADAMDEC1*	5.15	2.75	1.87
NM_001733	*C1R*	4.64	8.03	-1.73
NM_001710	*CFB*	3.20	5.93	-1.85
NM_002003	*FCN1*	3.01	3.16	-1.05
NM_080722	*ADAMTS14*	2.29	2.29	1.00
NM_002818	*PSME2*	2.15	2.00	1.07
**Phosphatases**				
NM_015568	*PPP1R16B*	4.22	6.21	-1.47
NM_152386	*SGPP2*	3.12	3.12	1.00
NR_036579	*APTX*	2.03	2.45	-1.21
**Other Enzymes**				
NM_004878	*PTGES*	31.71	36.95	-1.17
NM_001024465	*SOD2*	17.94	12.83	1.40
NM_002164	*IDO1*	12.19	12.19	1.00
NM_001134486	*GBP5*	12.01	10.60	1.13
NM_001037339	*PDE4B*	11.06	7.69	1.44
NM_000096	*CP*	10.38	12.60	-1.21
NM_000636	*SOD2*	9.43	6.19	1.52
NM_001024466	*SOD2*	7.81	4.84	1.61
NM_052941	*GBP4*	7.63	7.63	-1.00
NM_000161	*GCH1*	6.43	7.98	-1.24
NM_000265	*NCF1*	5.13	8.45	-1.65

NM_006041	*HS3ST3B1*	4.68	5.24	-1.12
NM_004273	*CHST3*	4.17	4.51	-1.08
NM_021615	*CHST6*	3.91	3.91	1.00
NM_001258041	*HARS*	3.90	2.53	1.54
NM_006290	*TNFAIP3*	3.85	2.11	1.82
NM_017594	*DIRAS2*	3.75	3.91	-1.04
NM_000127	*EXT1*	3.31	2.85	1.16
NM_182757	*RNF144B*	3.05	2.38	1.28
NM_004267	*CHST2*	2.72	2.29	1.19
NM_001161819	*MYO1B*	2.68	3.17	-1.18
NM_001001438	*LSS*	2.68	2.23	1.20
NM_000785	*CYP27B1*	2.67	2.30	1.16
NM_152363	*ANKLE1*	2.64	2.26	1.17
NM_001257231	*ALG13*	2.62	3.24	-1.24
NM_173701	*WARS*	2.52	3.73	-1.48
NM_001628	*AKR1B1*	2.41	3.02	-1.26
NM_000130	*F5*	2.35	3.36	-1.43
NM_025239	*PDCD1LG2*	2.27	2.32	-1.02
NR_037947	*NSUN2*	2.20	2.05	1.07
NM_001024024	*GCH1*	2.11	2.11	1.00
NM_014905	*GLS*	2.08	2.18	-1.05
NM_001145437	*LSS*	2.04	2.10	-1.03
NM_001142575	*IMPDH1*	-2.26	-2.06	-1.09
NM_001160230	*ADARB1*	-2.48	-3.03	1.22
NM_152463	*EME1*	-2.54	-2.05	-1.24
NR_004862	*TYW5*	-2.89	-3.63	1.26
NM_014298	*QPRT*	-3.32	-2.36	-1.41
NM_001135825	*RNF185*	-3.45	-2.02	-1.71
NM_006680	*ME3*	-3.90	-3.87	-1.01
NM_058248	*DNASE2B*	-3.97	-2.95	-1.35
NM_020686	*ABAT*	-4.25	-2.40	-1.77
**Transcription Regulators**				
NM_144590	*ANKRD22*	5.33	7.93	-1.49
NM_005238	*ETS1*	4.99	2.74	1.82
NM_001271606	*BASP1*	4.03	3.10	1.30
NM_030915	*LBH*	3.77	2.43	1.55
NM_005098	*MSC*	3.45	3.06	1.13
NM_002198	*IRF1*	3.45	3.72	-1.08
NM_005178	*BCL3*	3.39	2.29	1.48
NM_139266	*STAT1*	3.11	3.46	-1.11

NM_007315	*STAT1*	2.93	3.19	-1.09
NM_001195286	*IRF4*	2.68	2.62	1.02
NM_001198786	*POU2F1*	2.51	2.57	-1.02
NM_000246	*CIITA*	2.49	4.04	-1.62
NM_001144961.4	*NFKBIL1*	2.41	3.01	-1.25
NM_001018072	*BTBD11*	2.23	2.23	1.00
NM_013351	*TBX21*	2.19	2.56	-1.17
NM_006732	*FOSB*	2.16	2.13	1.02
NM_014323	*PATZ1*	2.11	2.40	-1.14
NM_005657	*TP53BP1*	-2.10	-3.36	1.60
NM_001099679	*TRIM32*	-2.16	-2.46	1.14
NM_001168280	*WWTR1*	-3.11	-2.45	-1.27
NM_001168388	*CITED2*	-3.99	-5.31	1.33
NM_001202429	*ASB2*	-4.47	-3.30	-1.36
**Transmembrane Receptors**				
NM_000878	*IL2RB*	9.85	5.38	1.83
NM_006378	*SEMA4D*	5.74	6.53	-1.14
NM_001767	*CD2*	4.50	4.01	1.12
NM_002185	*IL7R*	3.76	2.40	1.57
NM_002189	*IL15RA*	3.67	4.76	-1.29
NM_000733	*CD3E*	3.23	2.52	1.28
NM_001007033	*CLEC6A*	3.00	5.62	-1.88
NM_001256765	*IL15RA*	2.79	2.62	1.06
NM_006139	*CD28*	2.58	2.40	1.07
NM_004195	*TNFRSF18*	2.56	2.44	1.05
NM_014207	*CD5*	2.56	2.00	1.28
NR_028076	*SCARF1*	2.46	2.17	1.13
NM_001781	*CD69*	2.42	2.37	1.02
NM_000395	*CSF2RB*	2.17	2.69	-1.24
NM_001001389	*CD44*	2.15	2.60	-1.21
NM_173064	*IFNLR1*	-2.56	-2.95	1.15
**Transporters**				
NM_145640	*APOL3*	12.40	7.94	1.56
NM_178833	*SLC9B2*	10.33	5.76	1.79
NM_017585	*SLC2A6*	9.02	8.15	1.11
NR_027835	*APOL3*	6.43	5.59	1.15
NR_027833	*APOL3*	5.53	4.93	1.12
NM_001145099	*SLC2A6*	5.37	6.30	-1.17
NM_014331	*SLC7A11*	4.70	4.52	1.04
NM_013309	*SLC30A4*	4.16	6.19	-1.49

NM_033105	*DNAJC5B*	3.98	3.39	1.17
NR_037670	*SNX10*	2.95	2.90	1.02
NM_001199837	*SNX10*	2.06	2.60	-1.26
NM_001077241	*SLC25A45*	-2.18	-2.52	1.16
NM_145759	*TRAF5*	-2.25	-2.19	-1.03
NM_001270623	*SLC16A7*	-2.42	-2.66	1.10
NR_024195	*TOM1*	-2.45	-2.59	1.06
NM_001243013	*ABCB9*	-2.96	-2.61	-1.13
NM_033102	*SLC45A3*	-3.16	-2.87	-1.10
**Others**				
NM_015714	*G0S2*	25.73	18.89	1.36
NM_007115	*TNFAIP6*	11.78	20.37	-1.73
NM_178232	*HAPLN3*	9.89	9.89	1.00
NM_001190947	*TRAF1*	9.45	5.19	1.82
NM_001252392	*TNIP1*	8.96	5.32	1.68
NM_005951	*MT1H*	8.21	6.33	1.30
NM_001252391	*TNIP1*	7.73	4.89	1.58
NR_046450	*LOC374443*	7.56	5.45	1.39
NM_015549	*PLEKHG3*	7.54	6.83	1.10
NR_003187	*NCF1C*	7.33	9.09	-1.24
NM_001099287	*NIPAL4*	7.33	5.72	1.28
NM_031476	*CRISPLD2*	6.24	5.08	1.23
NM_005950	*MT1G*	6.19	6.03	1.03
NR_003186	*NCF1B*	5.59	10.53	-1.88
NM_001017995	*SH3PXD2B*	5.39	7.41	-1.37
NM_001130677	*C17orf96*	4.95	4.46	1.11
NR_022014	*HMGN2P46*	4.93	6.51	-1.32
NM_001111018	*NAV2*	4.54	3.01	1.51
NM_001169110	*SCO2*	4.48	6.23	-1.39
NM_001024858	*SPTB*	4.44	5.10	-1.15
NM_001193302	*SEMA4A*	4.35	3.09	1.41
NM_000607	*ORM1*	4.18	5.55	-1.33
NM_001244950	*SPOCK2*	3.88	2.91	1.33
NM_015393	*PARM1*	3.78	4.10	-1.08
NM_024430	*PSTPIP2*	3.77	4.69	-1.24
NM_001080421	*UNC13A*	3.77	3.87	-1.03
NR_038319	*LOC100505716*	3.69	2.96	1.24
NM_001244960	*FRMD3*	3.53	3.38	1.04
NR_026880	*MGC12916*	3.42	3.85	-1.13
NM_024508	*ZBED2*	3.33	3.06	1.09
NM_001252385	*TNIP1*	3.29	3.57	-1.09

NM_001258456	*TNIP1*	3.22	2.25	1.43
NM_182508	*FAM216B*	3.18	4.43	-1.39
NM_005953	*MT2A*	3.15	4.67	-1.48
NM_001270729	*BCL2L13*	3.10	3.01	1.03
NM_015111	*N4BP3*	3.08	2.64	1.17
NM_001243746	*FAM20A*	3.00	5.13	-1.71
NM_001258455	*TNIP1*	2.98	2.26	1.32
NM_022838	*ARMCX5*	2.91	2.53	1.15
NM_152866	*MS4A1*	2.83	3.78	-1.34
NM_198572	*SPATC1*	2.81	2.89	-1.03
NM_032413	*C15orf48*	2.70	2.77	-1.03
NM_001013838	*RLTPR*	2.69	2.03	1.32
NM_024873	*TNIP3*	2.61	2.61	1.00
NM_006779	*CDC42EP2*	2.52	2.50	1.01
NM_152512	*ENTHD1*	2.50	2.55	-1.02
NM_014398	*LAMP3*	2.50	2.22	1.13
NM_152320	*ZNF641*	2.43	3.94	-1.62
NM_175617	*MT1E*	2.43	3.39	-1.40
NM_001198812	*MSANTD3-TMEFF1*	2.42	2.86	-1.18
NM_031281	*FCRL5*	2.37	2.01	1.18
NR_003945	*GVINP1*	2.34	2.78	-1.19
NR_045116	*C5orf56*	2.32	2.20	1.06
NM_001260492	*RDX*	2.30	2.67	-1.16
NR_034033	*LOC285972*	2.25	2.51	-1.11
NM_001164440	*ANKRD33B*	2.25	2.85	-1.27
NR_024420	*LOC389634*	2.24	2.86	-1.28
NM_006080	*SEMA3A*	2.22	2.17	1.02
NM_001242348	*LOC100287177*	2.19	2.22	-1.01
NM_002353	*TACSTD2*	2.17	3.57	-1.64
NM_197955	*C15orf48*	2.10	2.25	-1.07
NM_001136199	*GRAMD1A*	2.04	2.27	-1.11
NM_020128	*MDM1*	2.03	2.17	-1.07
NR_033652	*LOC100132891*	2.01	2.21	-1.10
NM_152496	*MANEAL*	2.01	2.88	-1.44
NM_020153	*IFT46*	-2.02	-2.74	1.35
NM_024661	*CCDC51*	-2.03	-2.62	1.29
NR_003336	*SNORD116-22*	-2.04	-2.09	1.03
NR_023344	*RNU6ATAC*	-2.04	-2.05	1.00
NM_015122	*FCHO1*	-2.06	-2.41	1.17
NM_153436	*ODF2*	-2.10	-2.42	1.15

NM_001198690	*PPAN-P2RY11*	-2.10	-2.29	1.09
NR_003015	*SNORA53*	-2.14	-2.79	1.31
NM_198310	*TTC8*	-2.15	-2.93	1.37
NR_003317	*SNORD116-2*	-2.18	-2.02	-1.08
NR_002971	*SNORA38*	-2.20	-2.36	1.07
NR_002325	*SNORA6*	-2.22	-2.04	-1.09
NR_033579.1	*LOC440297*	-2.32	-2.51	1.08
NR_002964	*SNORA28*	-2.36	-2.05	-1.15
NM_001135812	*FAM60A*	-2.47	-2.41	-1.02
NR_003334	*SNORD116-20*	-2.56	-2.28	-1.12
NR_047529	*SIGLEC17P*	-2.61	-2.26	-1.15
NM_052820	*CORO2A*	-2.61	-2.29	-1.14
NM_001242948	*EPDR1*	-2.63	-3.45	1.31
NR_003026	*SNORA1*	-2.63	-2.30	-1.15
NR_003339	*SNORD116-25*	-2.74	-2.19	-1.25
NM_133168	*OSCAR*	-2.76	-3.29	1.19
NM_001001925	*MTUS1*	-2.94	-3.80	1.29
NR_073072.4	*NRM*	-2.96	-2.17	-1.37
NM_001168478	*ARMCX5*	-2.98	-2.44	-1.22
NM_001267042	*ARMC8*	-2.99	-3.09	1.03
NM_001202412	*ZNF559*	-3.01	-2.76	-1.09
NM_001143888	*BSDC1*	-3.13	-2.18	-1.44
NR_026759	*FAM95B1*	-3.15	-2.98	-1.06
NM_001166270	*HAUS4*	-3.45	-2.28	-1.51
NM_052924	*RHPN1*	-3.70	-2.16	-1.71
NM_199280	*FAM179A*	-3.74	-2.78	-1.34
NR_003009	*SCARNA8*	-4.01	-2.86	-1.40
NM_001134415	*TBCCD1*	-4.05	-2.51	-1.61
NM_020971	*SPTBN4*	-4.25	-2.51	-1.69
NM_001129727	*PLEKHG4*	-4.42	-2.94	-1.51
NM_001081004	*COMMD5*	-4.57	-2.60	-1.76

Supplementary Table 4. Signaling pathways that are uniquely modulated in Mob-MDM

	Activation z-score	
**Signaling Pathway**	**Mob-MDM vs GM-MDM and M-MDM**	**M-MDM vs GM-MDM**
Production of Nitric Oxide and Reactive Oxygen Species in Macrophages	2.45	-1.63
Acute Phase Response Signaling	2.45	0.82
Dendritic Cell Maturation	2.24	0.45
TREM1 Signaling	2.24	-0.45
Role of Pattern Recognition Receptors in Recognition of Bacteria and Viruses	2.00	1.00
p38 MAPK Signaling	2.00	1.00
Role of IL-17F in Allergic Inflammatory Airway Diseases	2.00	1.00
IL-6 Signaling	2.00	0.00

Supplementary Table 5. Predicted modulated upstream regulators of transcripts that are uniquely and significantly differentially expressed in Mob-MDM relative to M-MDM and GM-MDM

	Activation z-score	
**Upstream regulators**	**Mob-MDM vs GM-MDM and M-MDM**	**M-MDM vs GM-MDM**
lipopolysaccharide	6.61	1.75
TNF	5.77	1.18
NFkB (complex)	5.64	1.17
IL1B	5.11	0.27
poly rI:rC-RNA	4.99	0.65
IFNG	4.98	-0.92
Salmonella enterica serotype abortus equi lipopolysaccharide	4.85	1.02
phorbol myristate acetate	4.70	0.79
F2	4.37	0.47
IL6	4.24	-1.08
IL1A	4.10	0.58
IL12 (complex)	3.88	0.00
E. coli B4 lipopolysaccharide	3.86	1.45
IL18	3.86	0.32
E. coli B5 lipopolysaccharide	3.84	1.26
TLR4	3.77	0.03
TLR7	3.76	1.53
camptothecin	3.73	-0.18
IL1	3.70	0.26
RELA	3.66	1.46
TLR9	3.64	0.73
CD40LG	3.62	1.83
IL12 (family)	3.61	-0.12
APP	3.60	1.23
MYD88	3.54	1.01
IL2	3.50	0.38
TLR3	3.38	-0.01
IRF7	3.37	0.83
STAT1	3.37	-1.02
Tlr	3.36	1.05
salmonella minnesota R595 lipopolysaccharides	3.35	1.96
CSF2	3.28	1.60

ERK1/2	3.25	0.78
PDGF BB	3.25	0.99
BCR (complex)	3.24	1.50
Vegf	3.22	1.69
IL27	3.17	-1.54
NOD2	3.14	1.12
Interferon alpha	3.14	0.70
OSM	3.10	-1.34
IFNA2	3.08	-1.40
resiquimod	3.08	1.28
peptidoglycan	3.08	0.38
tretinoin	2.99	-1.17
CEBPB	2.97	0.05
Tnf (family)	2.95	1.00
IFI16	2.95	1.53
ionomycin	2.94	-0.22
CCL5	2.92	0.10
MIF	2.91	0.29
P38 MAPK	2.90	0.84
IL5	2.90	0.20
IKBKB	2.89	0.30
TGM2	2.88	-0.69
PTGS2	2.79	0.70
RIPK2	2.79	1.26
E. coli lipopolysaccharide	2.76	0.83
CHUK	2.76	-0.88
AGT	2.76	0.16
IL17A	2.75	1.68
TICAM1	2.74	-0.12
ECSIT	2.74	0.61
IL21	2.71	0.97
AGN194204	2.65	1.13
EIF2AK2	2.62	0.35
cigarette smoke	2.61	0.16
HMGB1	2.60	0.15
ANGPT2	2.60	-0.14
STAT4	2.59	-0.31
bryostatin 1	2.58	-0.51
SMARCA4	2.57	-0.58
EGF	2.57	0.20
ERK	2.57	-0.55

PIK3R1	2.57	-0.02
imiquimod	2.57	0.36
HGF	2.56	-0.04
TNFSF11	2.55	1.18
Fibrinogen	2.55	1.34
CpG ODN 1826	2.55	1.41
TYROBP	2.54	0.80
TCR	2.50	0.32
PRKCD	2.48	0.45
Jnk	2.46	0.79
ID2	2.45	1.63
cardiotoxin	2.45	1.63
SELPLG	2.45	0.45
MAP3K14	2.45	0.00
lipoarabinomannan	2.45	0.00
IFN alpha/beta	2.45	-0.45
hydrogen peroxide	2.45	0.85
mycophenolic acid	2.43	1.39
TRADD	2.43	0.90
Fcer1	2.43	0.39
IL17a dimer	2.43	1.31
CD14	2.42	0.65
3M-001	2.42	0.05
TLR2	2.42	0.31
PGF	2.41	1.30
ID3	2.41	0.93
CCL3	2.41	1.08
IL3	2.41	1.26
TLR8	2.40	1.48
MAPK14	2.40	0.86
PRL	2.40	1.59
TLR1	2.39	0.79
IL1R1	2.39	0.48
CpG ODN 1668	2.39	0.85
Ap1	2.38	0.51
FN1	2.37	0.89
paclitaxel	2.37	0.80
CYR61	2.36	1.09
Pam3-Cys	2.36	1.42
IL33	2.35	-0.24
N-acetylmuramyl-L-alanyl-D-isoglutamine	2.34	0.79

CEBPA	2.34	-0.82
Pam3-Cys-Ser-Lys4	2.34	1.54
hyaluronic acid	2.33	0.93
Mek	2.33	0.43
NFKB1	2.30	0.52
etoposide	2.25	-0.47
PPIF	2.24	1.00
RNASE1	2.24	0.45
IL23	2.24	0.00
C3	2.23	1.29
thapsigargin	2.22	1.21
IL6R	2.22	0.55
FOXL2	2.22	0.13
Gm-csf	2.21	1.17
3M-011	2.21	-0.39
RNASE2	2.21	0.30
REL	2.20	1.32
PF4	2.20	0.25
carrageenan	2.20	0.36
cytarabine	2.20	1.09
JAK2	2.20	-0.60
thioacetamide	2.20	0.33
TAC1	2.20	1.40
PARP1	2.20	-0.15
enterotoxin B	2.20	1.60
AIMP1	2.20	0.26
uric acid	2.19	0.74
RAC1	2.19	0.47
IRF8	2.19	1.13
Ifn gamma	2.19	1.41
tunicamycin	2.19	1.62
bromodeoxyuridine	2.18	0.14
stallimycin	2.18	0.14
IFNGR1	2.18	-0.83
NAMPT	2.18	0.63
TLR5	2.18	0.76
A23187	2.17	0.04
MALP-2s	2.17	1.22
Ifn	2.17	-1.95
F2RL1	2.17	0.02
hemozoin	2.16	1.48

forskolin	2.16	0.80
bleomycin	2.16	1.95
IRF5	2.15	0.54
galactosylceramide-alpha	2.15	0.73
EGR1	2.14	-0.19
CCL11	2.14	0.82
FOXO3	2.14	1.58
TNFRSF8	2.13	0.75
cisplatin	2.13	-0.07
CD40	2.12	-0.58
IL32	2.12	0.99
TRAF6	2.12	0.79
arsenic trioxide	2.11	-0.12
CpG oligonucleotide	2.08	1.83
TNFSF10	2.04	0.49
reactive oxygen species	2.01	0.90
di(2-ethylhexyl) phthalate	2.00	1.00
IL22	2.00	1.00
TMEM173	2.00	1.00
F3	2.00	1.00
Fc gamma receptor	2.00	0.58
TGFB3	2.00	0.00
motexafin gadolinium	2.00	0.00
N(2)-(gamma-D-glutamyl)-meso-2,2'-diaminopimelic acid	2.00	0.00
MAPK3	2.00	-1.00
BCL11B	2.00	-1.00
GATA1	2.00	-1.00
NfkB-RelA	2.00	0.09
ZFP36	-2.00	-1.01
fontolizumab	-2.00	-1.00
vitamin E	-2.00	-1.00
SOCS1	-2.00	0.64
15-deoxy-delta-12,14 -PGJ 2	-2.02	-0.85
VIP	-2.05	-1.16
Bay 11-7082	-2.11	-1.59
genistein	-2.12	-1.81
SFTPA1	-2.12	-1.37
wortmannin	-2.13	-0.20
ESR1	-2.14	-0.31
mir-146	-2.15	-0.83
DICER1	-2.16	0.30

AMPK	-2.17	1.02
caffeic acid phenethyl ester	-2.20	-0.49
BCL3	-2.20	-0.36
RPSA	-2.20	-0.60
PS-1145	-2.20	0.07
LRP1	-2.20	-0.69
Sn50 peptide	-2.20	-0.94
docosahexaenoic acid	-2.21	0.39
Sb202190	-2.21	0.01
IKZF1	-2.22	0.55
PPP2R5C	-2.24	-1.00
H89	-2.26	-0.66
aspirin	-2.29	0.17
geldanamycin	-2.31	1.20
actinomycin D	-2.39	-0.83
pyrrolidine dithiocarbamate	-2.40	-0.99
miR-146a-5p (and other miRNAs w/seed GAGAACU)	-2.42	0.51
AG490	-2.42	0.12
Alpha catenin	-2.43	-0.73
2-amino-5-phosphonovaleric acid	-2.45	-1.63
Igm	-2.56	-0.21
N-acetyl-L-cysteine	-2.58	-1.13
NS-398	-2.60	-0.02
MEOX2	-2.65	-1.63
Nr1h	-2.75	-0.07
dexamethasone	-2.75	1.64
simvastatin	-2.77	1.21
GFI1	-2.77	-1.29
troglitazone	-2.77	-0.70
epigallocatechin-gallate	-2.79	0.05
NR3C1	-2.88	-0.92
SP600125	-2.89	-0.48
miR-155-5p (miRNAs w/seed UAAUGCU)	-2.90	-1.25
PPARG	-2.90	-1.61
IL1RN	-2.90	0.14
Tgf beta	-2.94	0.57
cyclosporin A	-3.00	-1.46
etanercept	-3.13	0.11
PD98059	-3.24	-1.79
curcumin	-3.24	-1.46
LY294002	-3.24	-1.58

CD28	-3.31	-1.37
mir-21	-3.45	-0.39
SB203580	-3.59	-0.99
CD3	-3.60	-0.34
infliximab	-3.86	-0.90
IL10RA	-3.97	-0.90

Supplementary Table 6. Predicted modulated biological functions that are unique to Mob-MDM

	Activation z-score	
**Diseases and Bio Functions**	**Mob-MDM vs GM-MDM and M-MDM**	**M-MDM vs GM-MDM**
migration of cells	4.95	0.16
cell movement of leukocytes	4.84	0.65
cell movement	4.83	0.24
leukocyte migration	4.79	0.89
cell movement of blood cells	4.70	0.69
cell movement of myeloid cells	4.54	1.02
cell movement of mononuclear leukocytes	4.41	0.26
homing of cells	4.40	0.74
cellular homeostasis	4.33	-0.12
cell movement of lymphocytes	4.26	0.18
cell movement of phagocytes	4.24	0.81
chemotaxis	4.19	0.73
cell movement of lymphoid cells	4.11	0.31
homing of leukocytes	4.01	0.54
chemotaxis of myeloid cells	3.92	1.38
Lymphocyte migration	3.91	0.10
migration of mononuclear leukocytes	3.85	0.35
inflammatory response	3.84	0.63
chemotaxis of phagocytes	3.70	1.09
chemotaxis of leukocytes	3.66	0.71
homeostasis of leukocytes	3.65	-0.31
cell movement of T lymphocytes	3.62	-0.29
cell movement of granulocytes	3.60	0.83
activation of phagocytes	3.59	0.26
adhesion of mononuclear leukocytes	3.54	0.70
chemoattraction	3.52	0.82
T cell homeostasis	3.47	0.00
T cell migration	3.46	-0.42
T cell development	3.45	-0.01
homing of mononuclear leukocytes	3.44	-0.52
cell viability of blood cells	3.42	0.94
attraction of leukocytes	3.37	0.72
chemotaxis of granulocytes	3.34	0.95
cell viability of leukocytes	3.31	1.37

immune response of leukocytes	3.31	-0.11
adhesion of lymphocytes	3.27	1.31
cell movement of tumor cell lines	3.26	0.02
attraction of phagocytes	3.25	1.12
generation of cells	3.22	0.67
migration of phagocytes	3.21	1.57
chemoattraction of leukocytes	3.21	0.25
cell movement of monocytes	3.19	0.46
cellular infiltration by lymphocytes	3.16	-1.07
stimulation of cells	3.16	1.32
development of mononuclear leukocytes	3.16	0.31
immune response of cells	3.16	0.02
chemotaxis of neutrophils	3.15	0.73
cellular infiltration of blood cells	3.15	-0.56
cell movement of eosinophils	3.15	1.21
development of lymphocytes	3.15	0.28
cell movement of dendritic cells	3.14	1.16
chemotaxis of monocytes	3.11	-0.15
quantity of metal ion	3.10	0.15
recruitment of leukocytes	3.10	1.32
attraction of myeloid cells	3.10	1.06
chemotaxis of tumor cell lines	3.09	0.14
chemoattraction of phagocytes	3.08	0.79
cell movement of neutrophils	3.08	0.30
activation of antigen presenting cells	3.06	-0.60
cellular infiltration	3.06	-0.35
cellular infiltration by leukocytes	3.05	-0.43
activation of granulocytes	3.04	1.22
mobilization of Ca2+	3.04	0.33
chemotaxis of mononuclear leukocytes	3.04	-0.37
differentiation of blood cells	3.01	0.61
cell movement of antigen presenting cells	3.01	0.83
binding of cells	3.01	0.75
development of blood cells	3.00	0.31
differentiation of mononuclear leukocytes	3.00	0.79
quantity of Ca2+	2.98	0.33
homing of lymphatic system cells	2.96	-0.63
adhesion of T lymphocytes	2.94	1.19
cellular infiltration by mononuclear leukocytes	2.93	-0.75

cell movement of peripheral blood leukocytes	2.93	1.10
activation of dendritic cells	2.93	0.33
differentiation of leukocytes	2.93	0.48
chemoattraction of myeloid cells	2.93	0.68
quantity of metal	2.92	0.31
cellular infiltration by lymphoid cells	2.91	-0.85
development of lymphatic system	2.90	0.48
recruitment of phagocytes	2.88	0.83
quantity of blood cells	2.86	-1.20
homing of lymphocytes	2.83	-0.83
differentiation of lymphocytes	2.83	0.89
delayed hypersensitive reaction	2.79	0.94
mobilization of leukocytes	2.78	0.16
cell movement of PBMCs	2.77	0.82
attraction of mononuclear leukocytes	2.77	-0.06
polarization of cells	2.77	0.85
cell movement of hematopoietic cells	2.76	1.95
shape change of leukocytes	2.72	-0.14
activation of neutrophils	2.72	1.23
recruitment of myeloid cells	2.71	0.90
stimulation of leukocytes	2.71	1.06
development of lymphatic system cells	2.70	0.53
migration of myeloid cells	2.68	0.96
cell viability of lymphatic system cells	2.66	0.81
attraction of monocytes	2.65	0.38
migration of dendritic cells	2.64	1.01
cell viability of mononuclear leukocytes	2.64	0.87
response of myeloid cells	2.63	-0.20
recruitment of neutrophils	2.62	0.85
infiltration by T lymphocytes	2.61	-1.51
mobilization of myeloid cells	2.61	0.06
cell movement of natural killer cells	2.60	-0.69
mobilization of phagocytes	2.60	0.59
migration of neutrophils	2.60	0.27
polarization of blood cells	2.60	1.15
migration of granulocytes	2.59	0.70
atherosclerosis	2.58	-1.38
homing of T lymphocytes	2.58	-1.12
activation of blood cells	2.56	-0.35
differentiation of T lymphocytes	2.56	0.59

immune response of T lymphocytes	2.55	-0.80
binding of blood cells	2.55	0.50
migration of antigen presenting cells	2.54	1.48
metabolism of reactive oxygen species	2.52	0.51
activation of cells	2.50	0.28
chemotaxis of lymphatic system cells	2.50	-0.48
cell viability of lymphocytes	2.48	1.13
migration of pericytes	2.45	0.00
activation of leukocytes	2.43	-0.35
quantity of leukocytes	2.43	-1.04
attraction of antigen presenting cells	2.43	1.17
chemotaxis of PBMCs	2.43	1.07
NK cell migration	2.42	-0.27
priming of leukocytes	2.42	1.28
differentiation of lymphatic system cells	2.42	1.09
mobilization of neutrophils	2.42	0.63
response of myeloid leukocytes	2.42	-0.11
polarization of leukocytes	2.42	0.91
attraction of lymphocytes	2.41	-0.14
recruitment of granulocytes	2.41	0.87
chemoattraction of mononuclear leukocytes	2.40	-0.69
chemotaxis of eosinophils	2.39	0.11
synthesis of reactive oxygen species	2.39	0.42
quantity of lymphoid tissue	2.39	0.26
quantity of lymphatic system cells	2.39	-0.98
fever	2.38	0.42
cell viability of T lymphocytes	2.38	0.79
transmigration of mononuclear leukocytes	2.37	0.99
recruitment of mononuclear leukocytes	2.37	0.82
respiratory burst	2.36	0.84
attraction of granulocytes	2.36	0.51
quantity of cells	2.35	-0.86
experimental autoimmune encephalomyelitis	2.33	0.56
stimulation of mononuclear leukocytes	2.32	1.30
hypersensitive reaction	2.32	0.13
proliferation of cells	2.32	-0.19
binding of leukocytes	2.31	0.75
cell movement of macrophages	2.31	0.85
chemotaxis of T lymphocytes	2.29	-0.62
response of phagocytes	2.29	-0.45
growth of tumor	2.27	-0.79

differentiation of cells	2.26	1.48
NK cell proliferation	2.25	-0.91
quantity of natural killer cells	2.25	-0.63
cell movement of cytotoxic T cells	2.24	0.45
migration of hepatic stellate cells	2.24	-0.45
quantity of natural killer T lymphocytes	2.22	0.31
cellular infiltration of phagocytes	2.22	0.18
attraction of macrophages	2.22	1.11
chemotaxis of peripheral blood monocytes	2.22	1.00
chemotaxis of carcinoma cell lines	2.22	-1.39
binding of mononuclear leukocytes	2.22	-0.22
transendothelial migration of leukocytes	2.21	0.65
adhesion of endothelial cells	2.21	-0.02
binding of lymphatic system cells	2.21	0.08
polarization of mononuclear leukocytes	2.21	1.38
recruitment of antigen presenting cells	2.20	0.15
priming of lymphocytes	2.20	1.63
recruitment of monocytes	2.20	0.44
chemoattraction of lymphocytes	2.20	-0.53
response of neutrophils	2.20	-0.23
attraction of T lymphocytes	2.20	0.24
chemotaxis of antigen presenting cells	2.18	1.02
migration of Langerhans cells	2.18	1.11
recruitment of macrophages	2.16	0.39
attraction of neutrophils	2.15	0.89
chemoattraction of granulocytes	2.15	0.07
cell movement of naive lymphocytes	2.13	0.94
quantity of T lymphocytes	2.13	-0.02
quantity of lymphoid cells	2.12	-1.02
stimulation of T lymphocytes	2.12	1.26
Encephalitis	2.12	0.40
differentiation of helper T lymphocytes	2.12	0.40
accumulation of phagocytes	2.10	-0.85
quantity of mononuclear leukocytes	2.10	-1.31
formation of lymphoid tissue	2.09	0.08
response of mononuclear leukocytes	2.02	0.27
ion homeostasis of cells	2.02	0.03
quantity of lymphocytes	2.00	-1.18
migration of effector lymphocytes	2.00	1.00
chemoattraction of monocytes	2.00	0.00
priming of T lymphocytes	2.00	1.40

hypoplasia of lymph node	-2.45	0.00
hypoplasia of lymphoid organ	-2.55	-1.10
Parasitic Infection	-3.15	0.16
infection of mammalia	-3.26	0.57
